# Flexible Dual-Modal Sensing Transistor Enabled by Deep Learning Decoupling for Independent Light and Temperature Reconstruction

**DOI:** 10.1007/s40820-026-02285-7

**Published:** 2026-07-07

**Authors:** Shilin Lu, Ji Hoon Han, Dong Keun Lee, Sun Min Song, Huixin Yu, Sujin Jung, Lu Zhang, Zhao Yao, Jong Bin An, Hyun Jae Kim

**Affiliations:** 1 School of Electrical and Electronic Engineering, Yonsei University, Seoul, 03722 Republic of Korea; 2 Department of Integrated Display Engineering, Yonsei University, Seoul, 03722 Republic of Korea; 3 College of Electronic and Information, Qingdao University, Qingdao, 266071 People’s Republic of China; 4 BIT Micro Fab Research Center, Yonsei University, Seoul, 03722 Republic of Korea

**Keywords:** Flexible electronics, Light-temperature dual-modal sensing, Thin-film transistors, Deep learning, Wearable systems

## Abstract

Herein, a flexible dual-modal sensing transistor (FDST) is reported, based on zinc oxide nanofibers (ZnO NFs) integrated onto an indium–gallium–zinc–oxide thin-film transistor, and combined with a deep learning-based signal decoupling strategy. Defect-mediated subgap excitation and thermally activated interfacial potential modulation enable high sensitivity dual-modal responses, delivering a broadband photoresponsivity (R) up to 2.69 A W^−1^ and a temperature coefficient (${TC}$) of 0.071 °C^−1^. To enable reliable discrimination and simultaneous reconstruction of light and temperature, a multibias readout physically encodes the coupled stimuli into a high-dimensional current fingerprint, which is decoded by a lightweight multilayer perceptron. This synergistic approach enables accurate and independent reconstruction of light intensity and temperature, achieving coefficients of determination (*R*^2^) around 0.99. The FDST exhibits exceptional mechanical robustness under 10,000 bending cycles and severe bending (2 mm radius). Furthermore, a wearable system based on a low-power microcontroller demonstrates real-time monitoring with negligible cross-interference between optical and thermal modalities under uncontrolled outdoor conditions. This work establishes a general strategy for resolving cross-sensitivity, paving the way for robust and intelligent artificial perception systems.

**Supplementary Information:**

The online version contains supplementary material available at 10.1007/s40820-026-02285-7.

## Highlights

- A flexible dual-modal sensing transistor enabling simultaneous light and temperature detection was developed.
- The device exhibits a broadband responsivity of 2.69 A W^−1^, a temperature coefficient of 0.071 °C^−1^, and stable operation under severe bending deformation.
- A multibias electrical fingerprint combined with a lightweight neural network enables accurate reconstruction of light intensity and temperature for wearable environmental monitoring.

**Supplementary Information:**

The online version contains supplementary material available at 10.1007/s40820-026-02285-7.

## Graphical abstract

**Figure:**
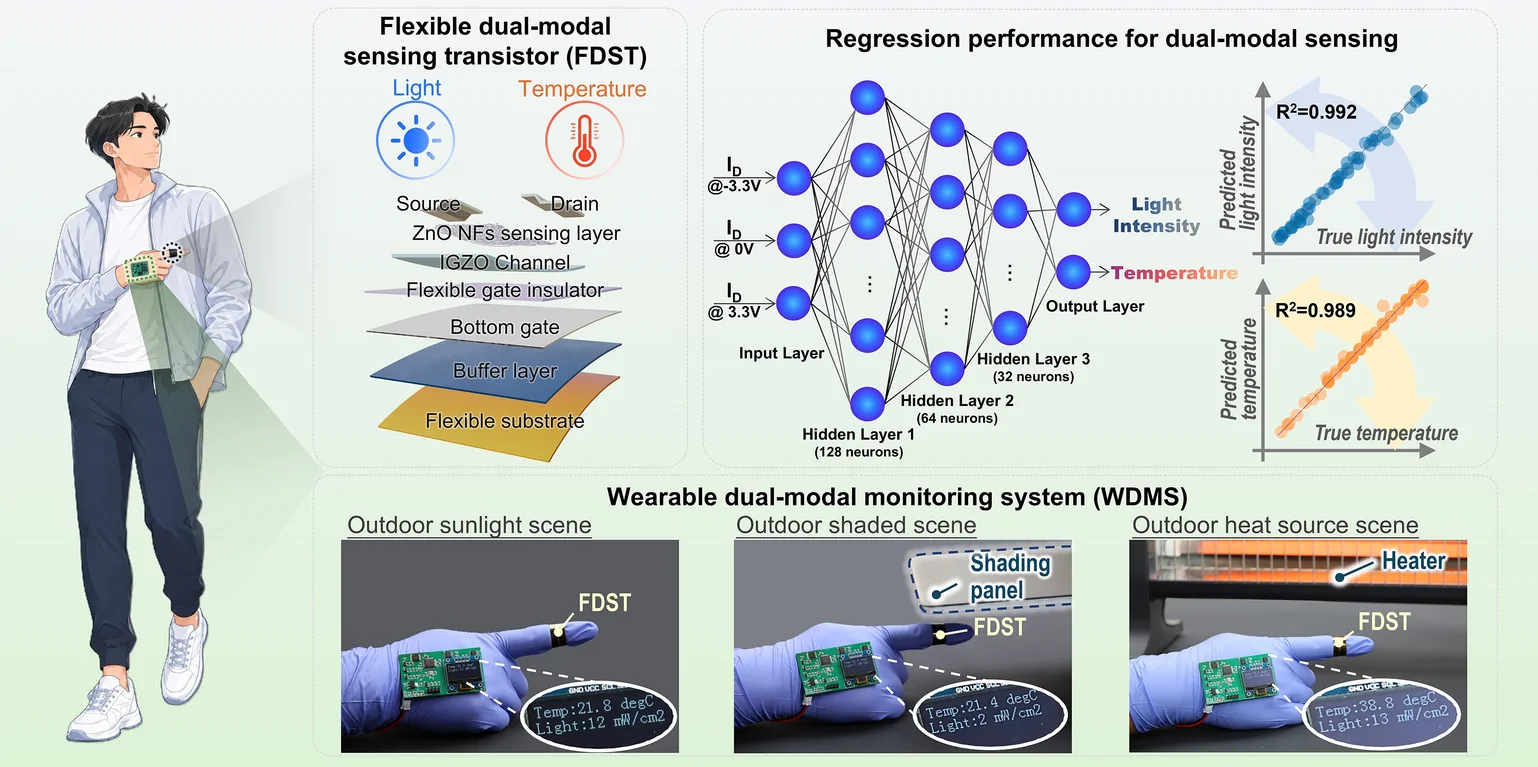

## Introduction

Multimodal perception is fundamental to both biological intelligence and artificial systems, enabling context-aware decision-making through the integration of diverse physical cues [1–7]. In natural organisms, light and thermal stimuli are perceived by distinct sensory pathways, namely the eyes and skin, and integrated by the brain to guide behavior [8, 9]. Mimicking this bimodal perception is particularly important in complex, unstructured environments where optical and thermal signals coexist, as their simultaneous and reliable acquisition underpins the fidelity of autonomous perception [10, 11]. Accordingly, with the rapid proliferation of electronic skins, wearables, and autonomous systems, the demand for such dual-modal information has encountered stringent engineering constraints, including device footprint, mechanical flexibility for conformal integration, and power consumption-driving a shift toward highly integrated sensing architectures [12–18].

**Fig. 1:**
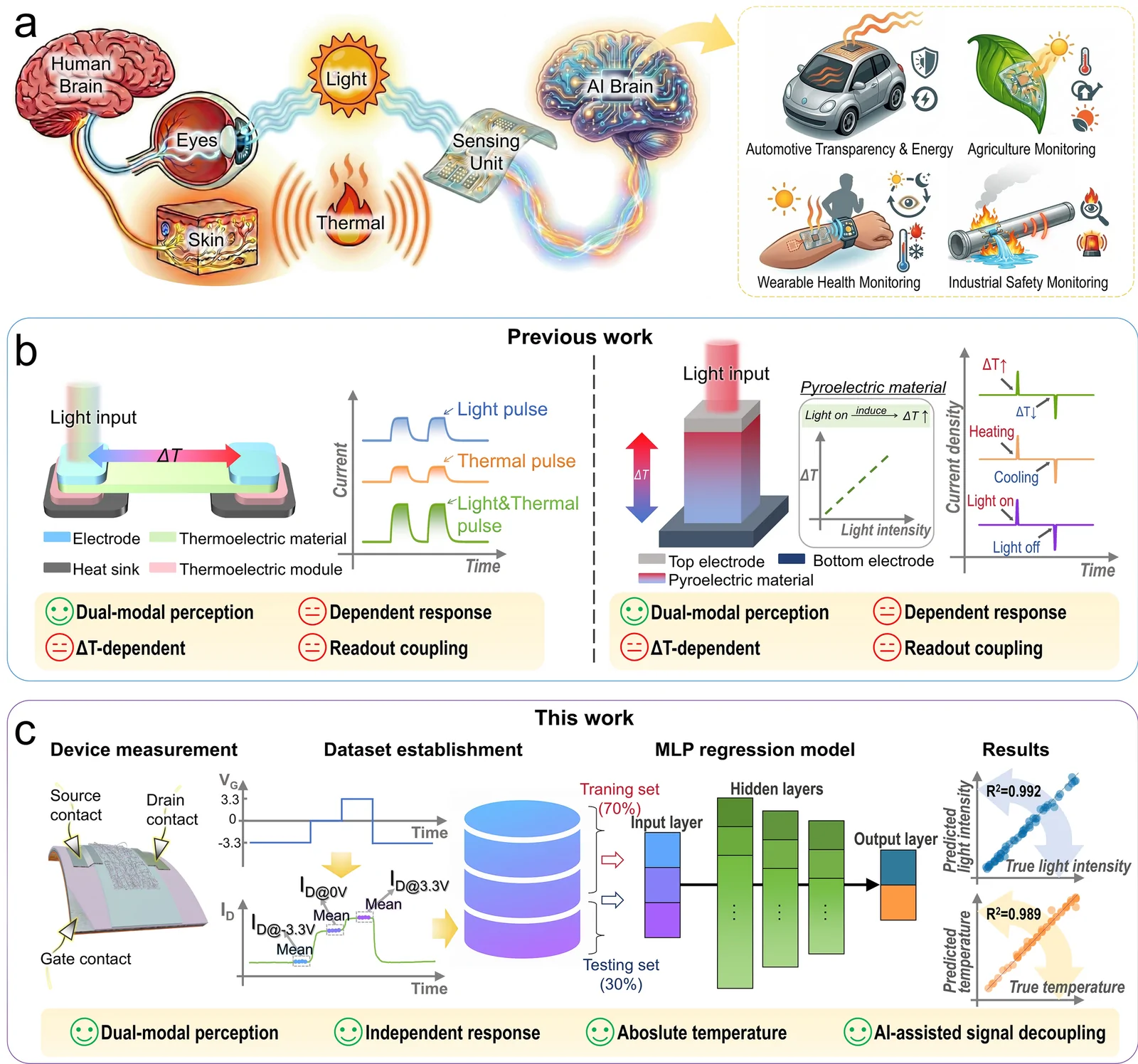
Design and implementation of the FDST. **a** Conceptual illustration of optical and thermal perception from biological systems toward integrated artificial sensing and intelligent applications. **b** Representative previous single-pixel devices for dual-modal light and temperature sensing based on thermoelectric effects (left, [28–30]) and pyroelectric effects (right, [31]). **c** This work on a flexible dual-modal sensing transistor for light and temperature sensing

By consolidating these functions, a single-unit photothermal sensing platform could synergistically optimize energy efficiency and transparency in smart automotive systems, support precision environmental monitoring in agriculture, facilitate health tracking in wearable electronics, and provide early warnings for safety–critical industrial scenarios, as schematically illustrated in Fig. 1a [19–22]. However, achieving simultaneous detection of light and temperature within a single sensing unit while maintaining reliable signal discrimination remains a critical challenge [23–27]. Accordingly, recent studies have predominantly focused on single-pixel multimodal devices based on thermoelectric effects [28–30] or pyroelectric effects [31], in which compact photothermal sensing is realized through integrated device architectures (Fig. 1b). Specifically, thermoelectric devices generate electrical outputs in response to temperature gradients (Δ*T*), whereas pyroelectric devices produce electrical signals through polarization effects induced by temperature changes, regardless of whether the heating originates from optical absorption or ambient thermal disturbances. In both cases, temperature sensing relies on variations rather than absolute temperature values. Consequently, although such devices exhibit dual-modal response capability, their optical sensing is inherently based on indirect photothermal transduction, lacking direct perception of optical information as an independent dimension. Moreover, because these mechanisms respond to dynamic thermal perturbations, such devices inherently lack the capacity to deliver accurate absolute temperature readings. Furthermore, the reliance on rigid material systems and gradient-dependent transduction mechanisms limits mechanical flexibility and hinders further device miniaturization, thereby restricting their broader applicability in deformable and wearable sensing scenarios.

In this work, we propose a flexible dual-modal sensing transistor (FDST) that enables simultaneous and distinguishable sensing of light and temperature within a single device (Fig. 1c). A nanostructured ZnO sensing layer is integrated onto an indium–gallium–zinc–oxide (IGZO) thin-film transistor (TFT) platform, and a multibias readout strategy is employed to encode the intertwined responses into bias-dependent electrical features [32–34]. These features are subsequently decoded using a lightweight multilayer perceptron (MLP) to reconstruct light intensity and absolute temperature. Beyond the device level, this study further extends to system-level multimodal perception by integrating the proposed FDST, the embedded wearable system, and the aforementioned decoupling model to construct a wearable dual-modal monitoring system (WDMS) [35–38].

## Experimental Section

### Device Fabrication

The flexible dual-modal sensing transistors were fabricated on a polyimide (PI) film laminated onto a silicon carrier wafer for mechanical support. To passivate the substrate surface and improve the mechanical durability against bending deformation, a 40-nm Al_2_O_*x*_ buffer layer was first grown onto the PI film via atomic layer deposition (ALD). Subsequently, a 100 nm Mo bottom gate was deposited on the Al_2_O_*x*_-buffered substrate via radio frequency (RF) magnetron sputtering. A mechanically durable HfO_*x*_/PTFE hybrid gate dielectric (~ 150 nm) was then deposited via co-sputtering, following the protocol reported in our previous work. For the channel layer, a 40-nm-thick IGZO film was deposited by RF magnetron sputtering. The IGZO sputtering target was composed of In_2_O_3_, Ga_2_O_3_, and ZnO at a 1:1:1 molar ratio. The active area was defined by a shadow mask, and the film was annealed at 300 °C to optimize carrier mobility. Mo source and drain electrodes were then sputtered through a shadow mask to complete the bottom-gate TFT structure. To fabricate the sensing layer, a precursor solution containing zinc nitrate hexahydrate (Zn(NO_3_)_2_·6H_2_O, 2.38 g) and PVP (Mw ≈ 1,300,000, 1 g) dissolved in a 7:3 (v/v) mixture of dimethylformamide (DMF) and ethanol was electrospun directly onto the channel. The as-spun precursor nanofibers were subjected to a two-stage thermal treatment: (i) preannealing at 200 °C in ambient air to facilitate solvent evaporation and partial decomposition of organic residues, followed by (ii) crystallization at 350 °C in a vacuum tube furnace. This vacuum treatment served a dual purpose: modulating the defect chemistry by generating essential oxygen vacancies for enhanced sensing performance, while simultaneously preserving the metallic integrity of the Mo electrodes. Finally, the device was mechanically delaminated from the silicon carrier, yielding a freestanding flexible sensor.

### Device Characterization

To investigate the surface morphology of the ZnO NFs before and after thermal annealing, atomic force microscopy (AFM, Dimension Edge; Bruker) was employed. Optical images of the fabricated devices were obtained using a digital microscope (VHX-7000; Keyence Corp.). The crystallinity of the ZnO films was determined by high-resolution XRD (SmartLab; Rigaku). The chemical compositions of the ZnO NFs and IGZO films were analyzed via XPS (PHI 5000 Versa Probe; Ulvac-PHI). The film thickness was measured using a spectroscopic ellipsometer (alpha-SE; J.A. Woollam). Furthermore, the electronic band structures and subgap states of the films were characterized using UPS (AXIS Supra; Kratos) and UV–Vis spectrophotometry (V-650; JASCO).

### Electrical Measurements

The electrical properties of the devices were evaluated using a parameter analyzer (4200A; Keithley Instruments). For the photoresponse measurements, a broadband white-light source was provided by a commercial high-power white LED module. The irradiance at the sample surface was calibrated using an optical power meter (PM100D; Thorlabs) prior to the electrical characterization. For temperature-dependent measurements, the device temperature was controlled by a hot chuck system (MST-1000B; MS TECH).

## Results and Discussion

### Device Structural Design and Material Characterization

**Fig. 2:**
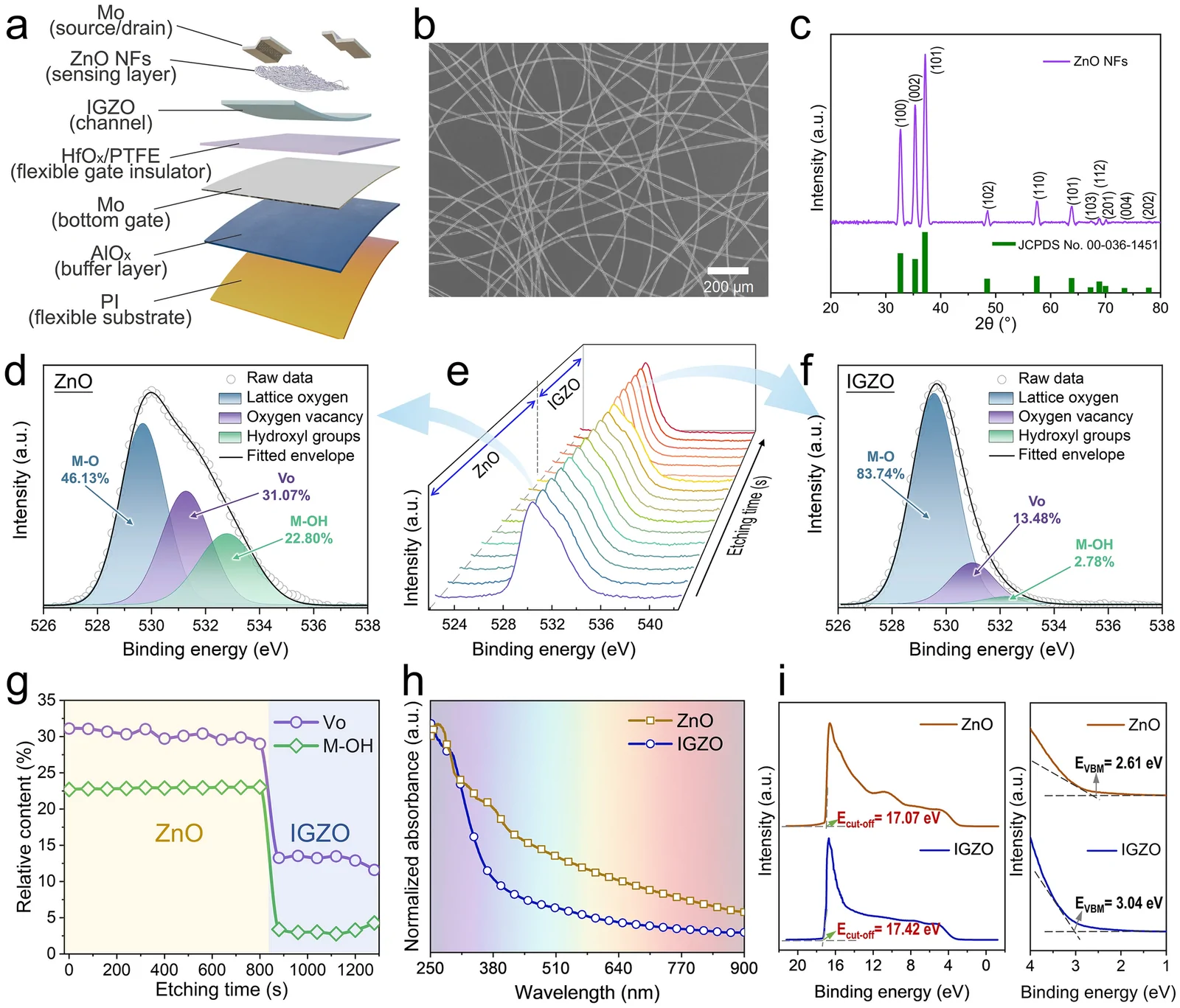
Structural architecture and material characterization of the FDST. **a** Schematic illustration of the FDST structure and layer stack. **b** Optical microscope image of the electrospun ZnO NFs network. **c** XRD pattern of the ZnO NFs. **d** Deconvoluted O 1*s* XPS spectrum of the ZnO sensing layer. **e** Depth-dependent O 1*s* XPS spectra across the ZnO/IGZO stack presented as a waterfall plot. **f** Deconvoluted O 1*s* XPS spectrum of the IGZO layer. **g** Relative content of V_O_ and M–OH as a function of etching time. **h** UV–Vis absorption spectra of ZnO and IGZO measured over the wavelength range of 250–900 nm after linear background subtraction and normalization. **i** UPS spectra of the ZnO and IGZO layers

The overall stacked structure, as shown in Fig. 2a, consists of a polyimide (PI) flexible substrate, an aluminum oxide (Al_2_O_*x*_) buffer layer, a molybdenum (Mo) bottom-gate electrode, a co-sputtered hafnium oxide–polytetrafluoroethylene (HfO_*x*_/PTFE) flexible gate insulator (GI) [39], an IGZO channel layer, a sensing layer composed of electrospun zinc oxide nanofibers (ZnO NFs), and Mo source/drain electrodes. The fabrication process is summarized in Fig. S1, with detailed procedures provided in the Experimental Section.

For the ZnO NFs sensing layer, electrospun nanofibers were employed to provide a high surface-to-volume ratio and enhanced environmental interaction, making their microstructural uniformity critical for stable sensing performance [40–43]. Fiber diameter distributions obtained under different electrospinning voltages are shown in Fig. S2, and the corresponding statistical results are summarized in Fig. S3 as mean ± standard deviation (SD). With increasing electrospinning voltage from 8 to 17 kV, the average fiber diameter decreases monotonically from 644.6 ± 111.3 to 547.7 ± 49.0 nm. Among all conditions, the fibers fabricated at 15 kV exhibit the smallest standard deviation (37.6 nm), compared to 49.0 nm at 17 kV. Although the average diameters at 15 and 17 kV are comparable, the diameter distribution at 15 kV is narrower and more symmetric, indicating improved structural uniformity of the ZnO NFs. When the voltage is further increased to 17 kV, a slight increase in diameter dispersion is observed, which is likely associated with jet instability and enhanced whipping behavior under an excessively strong electric field. Based on these results, and considering the improved structural uniformity, 15 kV was selected as the optimal electrospinning voltage for subsequent experiments. The effect of annealing treatment on fiber morphology was analyzed using atomic force microscopy (AFM), as shown in Fig. S4. The AFM height profiles indicate a clear morphological evolution after annealing, while the measured root-mean-square (RMS) roughness remains nearly unchanged. This morphological evolution is mainly attributed to the removal of residual solvents and organic components from the precursor, resulting in a stable inorganic fiber framework. Large-area optical microscopy (Fig. 2b) confirms that the electrospun ZnO NFs form a random yet continuous network under the optimized processing conditions. The ZnO NFs were characterized by X-ray diffraction (XRD), as shown in Fig. 2c. All diffraction peaks can be indexed to the hexagonal wurtzite phase of ZnO (JCPDS No. 36-1451), with no detectable impurity-related phases. An optimal electrospinning deposition time of 40 s is identified for the ZnO NFs (Fig. S5 and Note S1).

To resolve the spatial distribution of elements within the heterostructure, X-ray photoelectron spectroscopy (XPS) depth profiling was performed. The atomic concentration evolution of In, Ga, Zn, and O as a function of etching time is presented in Fig. S6. A sharp decrease in the Zn signal at approximately 800 s, accompanied by the emergence and stabilization of In and Ga signals, clearly identifies the transition from the ZnO nanofiber layer to the underlying IGZO channel. Based on the compositional transition identified from depth profiling, high-resolution O 1*s* spectra were extracted for the ZnO and IGZO regions. The O 1*s* spectrum of the ZnO layer (Fig. 2d) can be deconvoluted into three components centered at 530.0 ± 0.2, 531.0 ± 0.2, and 532.0 ± 0.2 eV, corresponding to lattice oxygen (M–O), oxygen vacancy (V_O_), and surface hydroxyl species (M–OH), with relative fractions of 46.13%, 31.07%, and 22.80%, respectively. In contrast, the O 1*s* spectrum of the IGZO layer (Fig. 2f) is dominated by lattice oxygen (83.74%), while V_O_ and M–OH components account for only 13.48% and 2.78%, respectively. This marked difference between the two regions reflects distinct chemical-state distributions across the heterostructure. The evolution of O 1*s* spectra during the etching process is shown in Fig. 2e. The spectral shape remains stable within the ZnO region and progressively shifts as the interface is approached, before stabilizing again in the IGZO region. The quantitative depth-dependent variation of oxygen-related components is summarized in Fig. 2g, where the fractions of V_O_ and M–OH decrease sharply after crossing the interface. The consistent trends observed in both spectral evolution and quantitative analysis further confirm the well-defined stratification of the ZnO/IGZO stack.

The optical absorption spectra of ZnO and IGZO are shown in Fig. 2h. For reliable spectral comparison, the spectra were processed by linear background subtraction followed by normalization prior to analysis. The corresponding Tauc plots derived from these spectra are presented in Fig. S7, from which the optical bandgaps were extracted as 2.71 and 3.57 eV, respectively. Ultraviolet photoelectron spectroscopy (UPS) was employed to determine the electronic energy levels (Fig. 2i). The secondary electron cutoff energies (*E*_cut-off_) are 17.07 eV for ZnO and 17.42 eV for IGZO. Using Φ = *hν* − E_cut-off_ (*hν* = 21.22 eV), the corresponding work functions are calculated to be approximately 4.15 and 3.80 eV, respectively. The valence band maxima (*E*_VBM_) relative to the Fermi level are 2.61 eV for ZnO and 3.04 eV for IGZO. By combining the UPS-derived parameters with the optical bandgaps obtained from Tauc analysis, the complete energy level alignment of the ZnO/IGZO heterojunction was constructed (Fig. S8).

### Optical Sensing Performance of the FDST

**Fig. 3:**
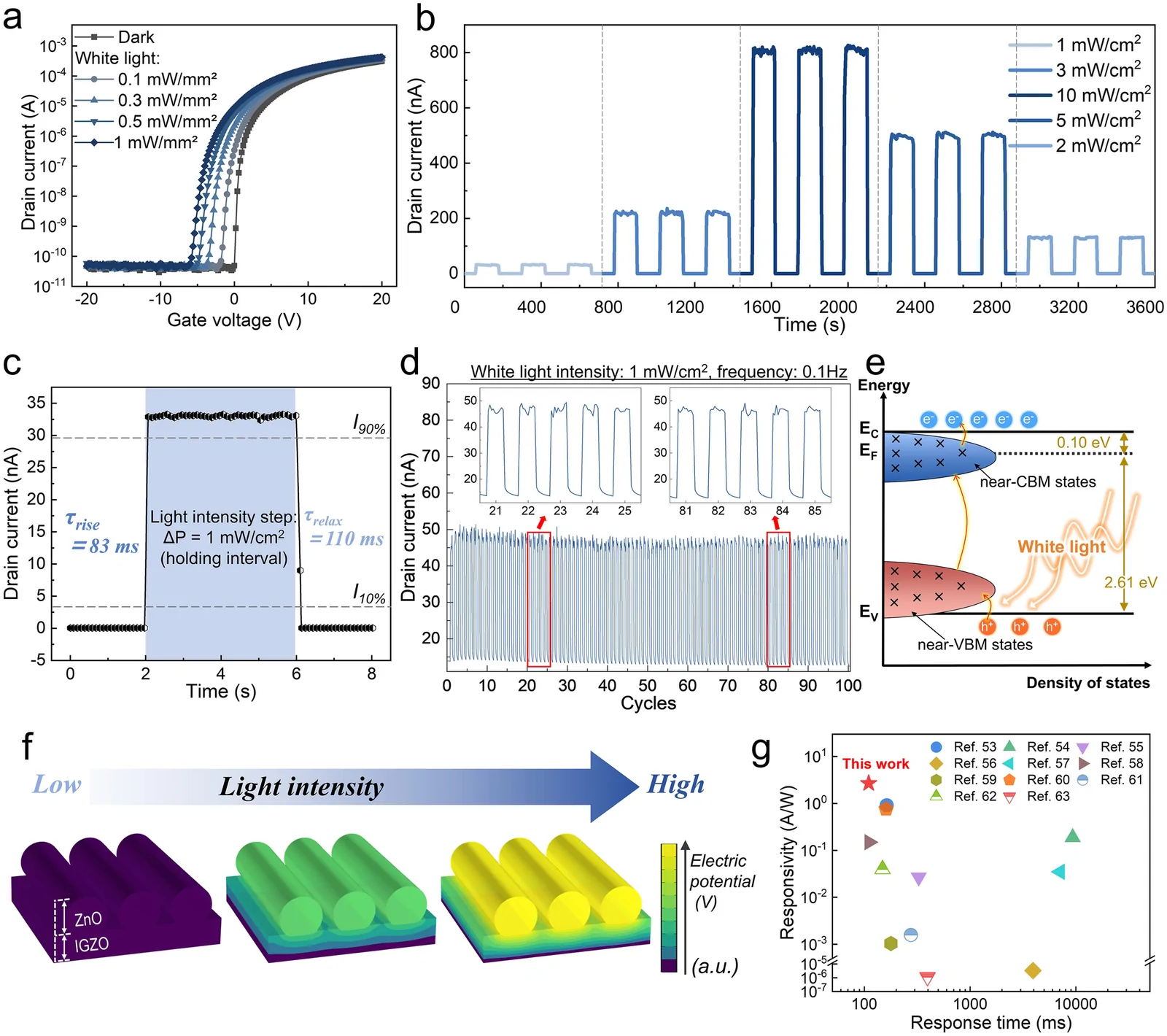
Optical sensing performance and mechanism of the FDST. **a** Transfer characteristics measured under different white-light illumination intensities. **b** Dynamic drain-current response under sequential light-intensity modulation. **c** Transient drain-current response to a single light on/off pulse. **d** Repetitive drain-current response under periodic white-light illumination, with insets showing enlarged cycles. **e** Schematic of subgap defect excitation and carrier trapping in ZnO under white-light illumination. **f** FEA-simulated potential distribution in the ZnO/IGZO stack as a function of light intensity. **g** Benchmark comparison of responsivity versus response time with representative reports

To clarify the role of the ZnO NFs sensing layer, a pristine IGZO TFT and a fabricated FDST are first presented in Fig. S9. Both devices share an identical layer structure and channel geometry, except for the presence of the ZnO NFs in the FDST. The channel length (*L*) and width (*W*) are 150 and 1000 μm, respectively. The electrical properties of the FDST were evaluated under dark conditions. The transfer curves of nine devices are shown in Fig. S10a, from which the average saturation mobility is 11.65 ± 0.24 cm^2^ V^−1^ s^−1^, the threshold voltage (*V*_th_) is 1.15 ± 0.18 V, the *I*_on_/*I*_off_ ratio is (1.35 ± 0.85) × 10^8^, and the subthreshold swing is 0.44 ± 0.01 V dec^−1^. Continuous cyclic testing for 3000 cycles (Fig. S10b) and dual-sweep measurement (Fig. S10c) were also performed to evaluate operational stability and hysteresis. The transfer curves remain largely overlapped during cycling, while the dual-sweep curves show negligible displacement. Control experiments were further conducted using a pristine IGZO TFT to identify the source of the multimodal response. As shown in Fig. S11a, the transfer characteristics of the pristine IGZO TFT measured under dark conditions and under white-light illumination with intensities ranging from 0.1 to 1 mW mm^−2^ are nearly identical, indicating negligible photoresponse in the absence of the ZnO sensing layer. In contrast, the FDST incorporating electrospun ZnO NFs exhibits pronounced light-dependent modulation (Fig. 3a). As the light intensity increases from dark to 1 mW mm^−2^, the transfer curves shift progressively toward negative gate voltage. This systematic shift confirms that optical excitation modulates channel conductivity through the ZnO sensing layer. To comprehensively quantify the photodetection performance, three key metrics were introduced: photoresponsivity (R), photosensitivity (S), and apparent specific detectivity (${D}_{\mathrm{a}\mathrm{p}\mathrm{p}}^{*}$) [44–46]. Under broadband white-light illumination, R and S are defined as:

1 $$R = \frac{{I_{{{\mathrm{light}}}} - I_{{{\mathrm{dark}}}} }}{{P_{{{\mathrm{in}}}} \cdot A}}$$

2 $$S = \frac{{I_{{{\mathrm{light}}}} }}{{I_{{{\mathrm{dark}}}} }}$$

Here, $I_{{{\mathrm{light}}}}$ and $I_{{{\mathrm{dark}}}}$ denote the drain current under illumination and in the dark, respectively; $P_{{{\mathrm{in}}}}$ is the incident white-light power density; A is the effective illuminated area, which was defined by a physical aperture mask exposing only the FDST channel region. Based on these definitions, the FDST exhibits a maximum broadband R of 2.69 A W^−1^ in the saturation regime, reflecting efficient photocarrier generation and collection. Under weak illumination at gate voltage (*V*_G_) = 0 V, the S reaches 6.96 × 10^3^, ensuring a high signal-to-noise ratio in optical signal readout. To evaluate the noise-limited detection capability, the dark-current fluctuation was experimentally characterized at a representative operating condition (*V*_G_ = − 0.1 V). As shown in Fig. S12, the time-domain dark-current traces exhibit stable fluctuations without noticeable drift, and the corresponding histograms follow Gaussian-like distributions, indicating stationary noise behavior. The root-mean-square (RMS) noise current was extracted from the steady-state current fluctuation, defined as:

3 $$i_{{{\mathrm{rms}}}} = \sqrt {\frac{1}{N - 1}\mathop \sum \limits_{i = 1}^{N} \left( {I_{i} - \overline{I}} \right)^{2} }$$

where $\overline{I}$ is the average current over the selected steady-state time window. The $D_{{{\mathrm{app}}}}^{*}$ can thus be written as:

4 $$D_{{{\mathrm{app}}}}^{*} = \frac{{R\sqrt {AB} }}{{i_{{{\mathrm{rms}}}} }}$$

where B is the effective noise bandwidth. Based on the experimentally extracted parameters summarized in Table S1, the FDST exhibits an apparent specific detectivity of 1.1 × 10^8^ Jones. Following recent guidelines for photodetector evaluation, this value is reported as ${D}_{\mathrm{a}\mathrm{p}\mathrm{p}}^{*}$, as it is derived from time-domain noise analysis rather than a rigorous frequency-domain noise characterization or an evaluation based on noise equivalent power [46]. The light-intensity-resolving capability was examined under stepwise and mixed-intensity sequences (Fig. 3b). Distinct steady-state current plateaus are observed and correlate with the incident white-light intensity. Notably, when high-intensity pulses are inserted into a sequence with random intensity fluctuations, the current rapidly returns to the steady-state level corresponding to the subsequent lower intensity without discernible hysteresis or residual memory. The transient response under a single light-intensity step is shown in Fig. 3c. The rise time (*τ*_rise_) and relax time (*τ*_relax_) are defined as the durations required for the current to transition between 10 and 90% of the response amplitude [44, 45]. These two key threshold levels are defined as follows:

5 $${I}_{90\%}={I}_{\mathrm{m}\mathrm{i}\mathrm{n}}+\left({I}_{\mathrm{m}\mathrm{a}\mathrm{x}}-{I}_{\mathrm{m}\mathrm{i}\mathrm{n}}\right)\times 0.9$$

6 $${I}_{10\%}={I}_{\mathrm{m}\mathrm{i}\mathrm{n}}+\left({I}_{\mathrm{m}\mathrm{a}\mathrm{x}}-{I}_{\mathrm{m}\mathrm{i}\mathrm{n}}\right)\times 0.1$$

Here, ${I}_{\mathrm{m}\mathrm{a}\mathrm{x}}$ and ${I}_{\mathrm{m}\mathrm{i}\mathrm{n}}$ represent the steady-state current after stimulus application (maximum response) and the initial baseline current, respectively. Based on this definition, the FDST exhibits rapid current transitions when illumination is switched on or off: *τ*_rise_ and *τ*_relax_ are as short as 83 and 110 ms, respectively. Long-term cycling stability was evaluated under a white-light intensity of 1 mW cm^−2^ at a switching frequency of 0.1 Hz (Fig. 3d). Quantitative analysis based on the extracted current values reveals that the average on-state current decreases by only 2.8% after 100 cycles, indicating highly stable and reproducible optical switching behavior. The enlarged views of representative early and late cycles further confirm the nearly identical waveform characteristics, demonstrating the robust operational stability of the device. The optical-response mechanism is illustrated in Fig. 3e. Oxygen-vacancy-related subgap defect states in ZnO introduce localized levels within the bandgap, enabling visible-light absorption below the intrinsic bandgap energy [47–49]. To further support this defect-mediated interpretation, the spectral distribution of the white LED used in Fig. 3a is provided in Fig. S13, confirming broadband emission in the visible range. In addition, wavelength-dependent transient photocurrent responses under monochromatic illumination at 405, 532 and 635 nm are presented in Fig. S14. Clear and reversible responses are observed at all three wavelengths, consistent with defect-assisted subgap excitation in the ZnO NFs sensing layer. Under illumination, photogenerated holes are preferentially trapped by deep defect states and act as fixed positive charge centers, elevating the local electrostatic potential of the ZnO layer [50, 51]. Through capacitive coupling across the ZnO/IGZO interface, this light-induced surface potential effectively acts as an additional internal gate bias. Specifically, the accumulation of trapped positive charges lowers the potential barrier for electron transport in the underlying IGZO channel, leading to the observed negative *V*th shift rather than a direct increase in bulk channel conductance. This photogating-dominated response is consistent with the parallel shift of the transfer curves and the nearly constant on-state drain current observed in Fig. 3a [52]. Finite element analysis (FEA) was conducted to simulate the light-intensity-dependent potential distribution within the ZnO/IGZO stack (Fig. 3f). In the model, light-induced surface-charge modulation was applied exclusively to the ZnO layer, and the resulting electrostatic potential was self-consistently propagated to the underlying IGZO channel through capacitive coupling. The simulation solves the Poisson equation with surface-charge boundary conditions, and the detailed governing equations and phenomenological descriptions are provided in Table S2 and Note S2. Using a saturation-type light-dependent surface-charge model, the simulated potential maps show a monotonic increase in surface and interfacial potential with increasing illumination intensity, consistent with the experimentally observed light-induced conductivity enhancement. To benchmark the overall white-light photodetection performance, responsivity versus response time is compared with representative white-light photodetectors reported in the literature (Fig. 3g and Table S3) [53–63]. The FDST resides in a region characterized by a balanced combination of high responsivity and relatively fast response dynamics.

### Thermal Sensing Performance of the FDST

**Fig. 4:**
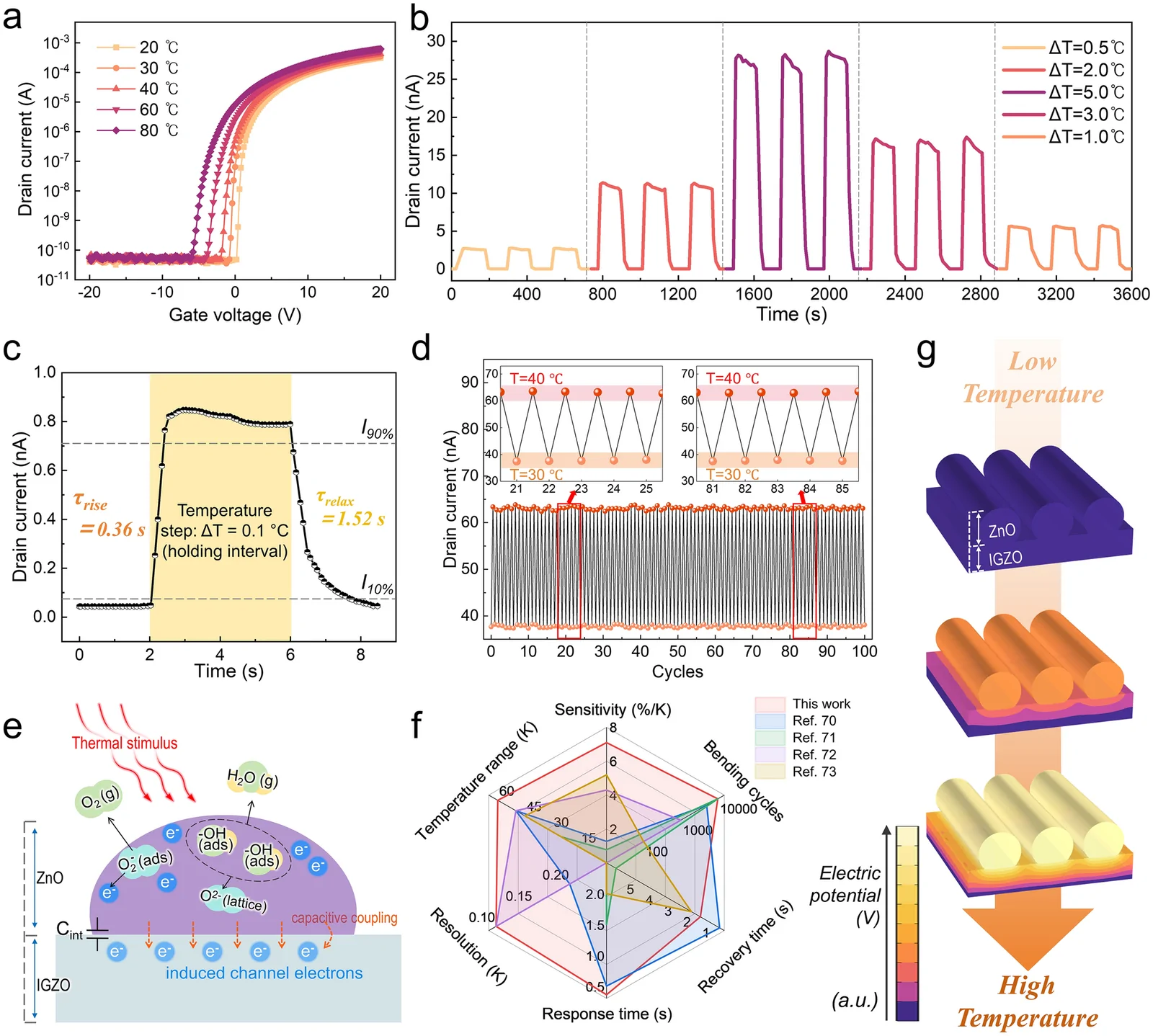
Thermal sensing performance and mechanism of the FDST. **a** Transfer characteristics measured under different temperatures. **b** Dynamic drain-current response under sequential temperature steps. **c** Transient drain-current response to a single-step thermal pulse. **d** Cycling stability under repeated temperature switching; insets show representative cycles. **e** Schematic illustration of thermally activated surface processes and capacitive coupling between ZnO and the IGZO channel. **f** Radar comparison of thermal sensing metrics among representative flexible temperature sensors. **g** FEA-simulated temperature-dependent potential distribution in the ZnO/IGZO stack

The temperature-dependent electrical characteristics of the FDST were systematically examined over the range of 20–80 °C. For comparison, a pristine IGZO TFT without ZnO nanofiber integration was measured under identical conditions. As shown in Fig. S11b, the transfer curves of the reference device remain nearly unchanged across the entire temperature range, indicating that the IGZO channel itself exhibits negligible intrinsic thermal sensitivity. In contrast, the FDST displays a clear and continuous modulation of its transfer characteristics with increasing temperature. As shown in Fig. 4a, the transfer curves shift progressively toward negative gate voltage, accompanied by a monotonic increase in the turn-on current. This systematic evolution reflects an enhancement of channel conductivity induced by thermal stimulation. We employ the temperature coefficient (TC) to quantify the device’s thermal response, defined as:

7 $$ {TC}=\frac{\Delta I/{I}_{0}}{\Delta T}\times 100\%$$

where ${I}_{0}$ is the drain current at the initial temperature, ΔI represents the temperature-induced current variation, and ΔT is the corresponding temperature change [64]. Within the tested range, the FDST exhibits a TC of 0.071 °C^−1^ in its typical operating state. Dynamic tests under sequential temperature modulations (Fig. 4b) demonstrate the device’s ability to distinguish varying thermal gradients, where the current plateaus accurately correspond to specific temperature increments (Δ*T* from 0.5 to 5.0 °C). The stepwise temperature response (Fig. 4c) further confirms the thermal tracking capability of the FDST. Using the same criteria defined in Eqs. (5) and (6), the FDST maintains a second-level response speed (*τ*_rise _≈ 0.36 s; *τ*_relax _≈ 1.52 s), despite the inherently slower ionic relaxation involved in thermochemical processes, enabling it to effectively capture rapid fluctuations in ambient temperature fields. Long-term stability under repeated thermal switching is shown in Fig. 4d, where the averaged steady-state drain current for each cycle is plotted over 100 cycles between 30 and 40 °C. The FDST exhibits negligible degradation, with the relative variation remaining within 1.5% and no observable monotonic decay trend. Enlarged views further confirm nearly unchanged amplitude and waveform shape, demonstrating excellent cyclic stability. The thermally induced interfacial modulation mechanism is illustrated in Fig. 4e. In the initial state, the ZnO surface is rich in adsorbed oxygen species (${\mathrm{O}}_{2}^{-}$) and hydroxyl groups (–OH), which introduce electron trapping on the surface of the n-type semiconductor. The resulting negatively charged surface layer establishes an electrostatic potential that induces upward band bending and electron depletion in the IGZO channel through capacitive coupling, thereby suppressing the dark-state conductivity [65]. Upon thermal stimulation, surface adsorption–desorption processes are activated on the ZnO surface. In particular, the desorption of surface-adsorbed oxygen species releases previously trapped electrons back into the conduction band [66–68], as described below:

8 $${\mathrm{O}}_{2}^{-}\left(\mathrm{ads}\right)\rightleftharpoons {\mathrm{O}}_{2}\left(\mathrm{g}\right)+{\mathrm{e}}^{-}$$

where ads and *g* denote the adsorbed and gaseous states, respectively. As the ${\mathrm{O}}_{2}^{-}$ species desorb, the electron depletion layer at the surface becomes thinner, leading to increased carrier concentration. Meanwhile, surface –OH undergoes a dehydration condensation reaction. Driven primarily by thermal activation, this dehydroxylation process is enabled by the thermal energy supplied at elevated temperatures, which facilitates the removal of surface hydroxyl groups and the healing of surface *V*_O_ [69]. The reaction kinetics are described as follows:

9 $$2{\mathrm{OH}}^{ - } \left( {{\mathrm{ads}}} \right) \rightleftharpoons {\mathrm{H}}_{2} {\mathrm{O}}\left( {\mathrm{g}} \right) + {\mathrm{O}}^{2 - } \left( {{\mathrm{lattice}}} \right)$$

The ${\mathrm{O}}^{2-}$ generated in the forward reaction refills *V*_O_ at the surface, is electrostatically compensated by the lattice field, and no longer acts as an additional negative charge center. The reduction in surface negative charge density leads to a gradual flattening of the upward band bending and a positive shift in the interfacial electrostatic potential, which is capacitively coupled to the IGZO channel as a temperature-dependent electrical modulation. When the heat source is removed (cooling) or the device returns to its initial equilibrium state, the above reactions proceed in reverse, leading to the reformation of the surface depletion layer and endowing the device with good reversibility. Figure 4f, with detailed data listed in Table S4, presents a six-dimensional comparison between the FDST and representative flexible temperature sensors [70–73], encompassing sensitivity, response and recovery times, resolution, temperature range, and bending cycles. Rather than excelling in a single parameter, the FDST maintains competitive performance across all metrics, highlighting its multiparameter optimization. As the thermal response is closely related to surface adsorption–desorption processes, the influence of ambient humidity (relative humidity, RH) was further evaluated. As shown in Fig. S15a–c, the transfer characteristics measured at 20, 40, and 60 °C under different RH levels (30%–70%) exhibit only minor variations, including a slight positive shift in threshold voltage and a marginal reduction in on-current as RH increases from 30% to 70%. The dynamic thermal responses under repeated temperature cycling (Fig. S15d) further demonstrate that the waveform shape and reversibility are well preserved across different RH conditions, with only a slight reduction in response amplitude observed at higher humidity. The $\it {TC}$ as a function of RH (Fig. S15e) shows a gradual decrease with increasing humidity. Notably, the $\it {TC}$ at 80% RH exhibits a maximum reduction of approximately 13.7% compared to that at 30% RH. These results suggest that moisture introduces a bounded perturbation rather than significantly disrupting the thermally activated sensing mechanism. Finally, the temperature-dependent potential evolution was examined by FEA (Fig. 4g). Thermal modulation was introduced at the ZnO surface through a temperature-dependent surface-charge model, and the induced electrostatic variation was subsequently transferred to the IGZO channel via interfacial capacitive coupling. The governing equations and boundary conditions are summarized in Table S2 and Note S2, while the main text focuses on the agreement between simulation and experimental observations.

### Mechanical Robustness and Response Stability under Dynamic Deformation

To evaluate the mechanical tolerance and operational reliability of the FDST under dynamic deformation, systematic flexibility tests were performed using a customized bending platform (Fig. S16a). Figure S16b depicts the transfer characteristics measured before and after 10,000 bending cycles at a small bending radius of 2 mm under three combined photothermal conditions: darkness at 20 °C, 0.1 mW mm^−2^ at 40 °C, and 0.3 mW mm^−2^ at 60 °C. Notably, even after extensive mechanical fatigue, the on-current, subthreshold region, and off-state noise floor exhibited negligible degradation, demonstrating the stable operation of the FDST for long-term operation. Beyond basic electrical survival, we further evaluated whether mechanical strain perturbs the dual-modal sensing outputs. Under tensile bending (Fig. S17a), the maximum R (Max.R) maintains a narrow range of 2.65–2.70 A W^−1^ across the entire bending-radius range (from 7 to 1 mm). The $\it {TC}$ also remained stable (0.068–0.071 °C⁻^1^) without discernible drift. A consistent robustness was observed under compressive bending (Fig. S17b), where Max. R (2.64–2.71 A W^−1^) and $\it {TC}$ (0.069–0.071 °C^−1^) exhibit only minor and reversible variations with curvature. To further examine the device morphology after repeated mechanical deformation, enlarged optical micrographs were recorded before and after 10,000 bending cycles (Fig. S18). No observable cracks or disruption of the ZnO nanofiber network were detected within the imaged region. These results collectively indicate that the FDST maintains stable photothermal transduction with limited sensitivity to mechanical deformation. Such robustness supports reliable dual-modal sensing under conditions relevant to flexible and wearable applications. To place the demonstrated performance in the context of existing multimodal sensing technologies, a quantitative comparison across key metrics is summarized in Table S5.

### Multibias Electrical Fingerprinting for Stimulus Reconstruction

**Fig. 5:**
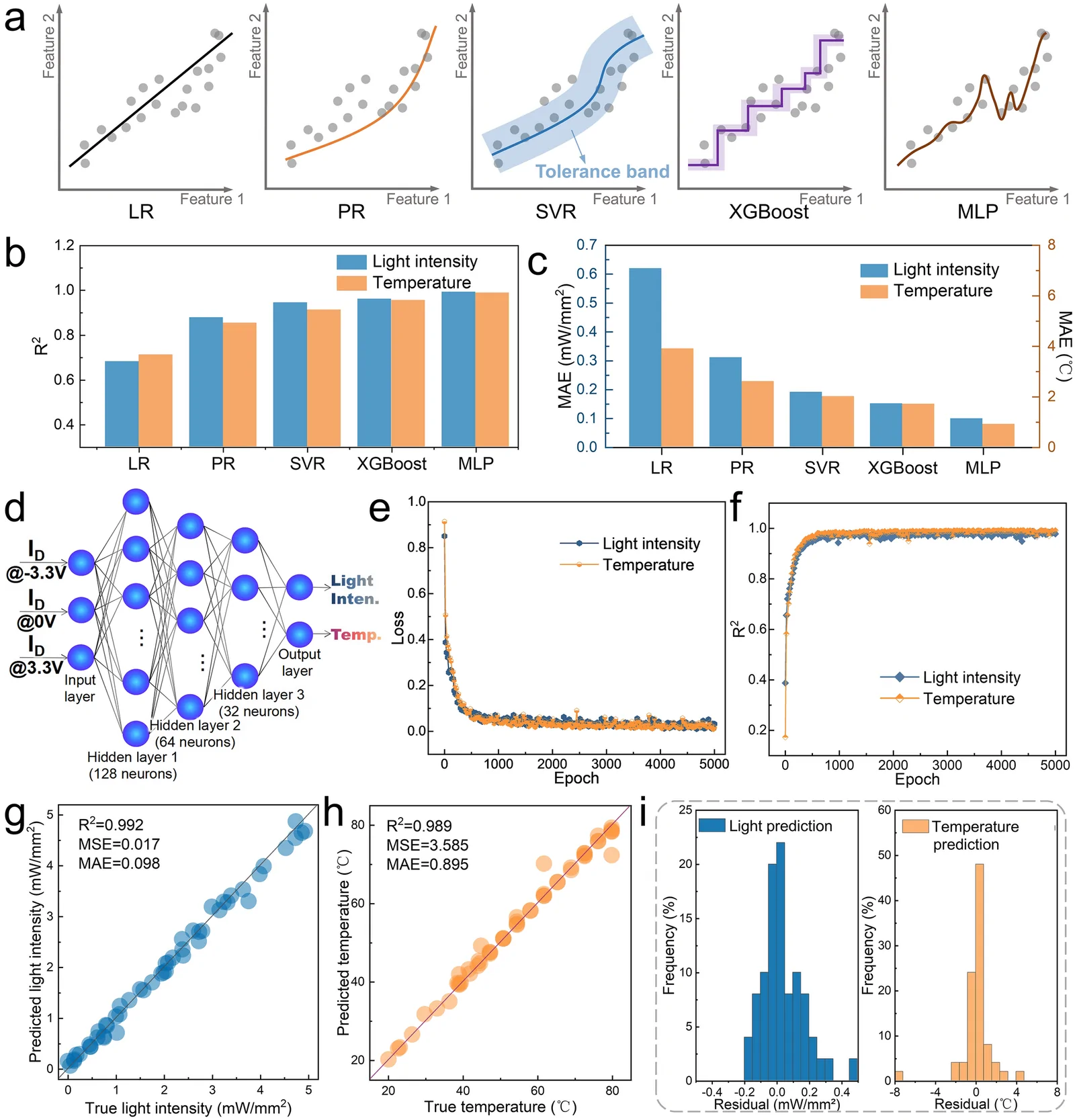
Evaluation and implementation of regression models for decoupled light intensity and temperature sensing. **a** Schematic comparison of regression algorithms, including LR, PR, SVR, XGBoost, and MLP. **b** *R*^2^ for light intensity and temperature prediction across models. **c** MAE for light intensity and temperature prediction across models. **d** Architecture of the MLP. **e** Training loss as a function of epoch for the MLP. **f** Evolution of *R*^2^ during MLP training. **g** Parity plot of predicted versus true light intensity using the MLP. **h** Parity plot of predicted versus true temperature using the MLP. **i** Residual distributions for MLP-based light intensity and temperature predictions

Although the FDST exhibits pronounced conductance modulation under both optical and thermal stimuli, the drain current measured at a single gate bias does not provide a bijective mapping to the stimulus pair. As evidenced in Fig. S19, distinct light–temperature combinations may generate nearly identical current values at a specific bias despite corresponding to fundamentally different physical states. This degeneracy arises because the optical and thermal perturbations, though governed by distinct physical mechanisms, are projected onto a one-dimensional current axis where their responses partially overlap. To eliminate the intrinsic ambiguity associated with single-point sampling, we construct a three-bias readout vector *X* = [*I*_*D*_ (− 3.3 V), *I*_*D*_ (0 V), *I*_*D*_ (+ 3.3 V)], which serves as a high-dimensional electrical fingerprint encoding the coupled optical–thermal response. These three gate biases were selected because they sample distinct regions of the transfer characteristics, exhibiting different sensitivities to optical and thermal perturbations. A detailed justification of this bias selection, together with a systematic ablation study over different bias combinations, is provided in Note S4 and Table S6, which confirms that the selected triplet achieves the optimal trade-off between decoupling accuracy and hardware constraints. This multibias strategy effectively transforms the modal coupling problem into a feature-separable representation problem, thereby rendering systematic model-based decoupling feasible. Recent IGZO-based sensing systems have increasingly incorporated machine learning and high-dimensional response engineering to enhance intelligent sensing and signal recognition. As summarized in Table S7, representative approaches include visual–tactile perception using IGZO TFTs integrated with an external triboelectric nanogenerator (TENG) [74], optoelectronic associative learning and neuromorphic pattern recognition in charge-trapping a-IGZO TFTs [75], optical-response-fingerprint-assisted gas recognition [76], and multidimensional gas discrimination based on complementary response signals from *p*-Cu_2_O/n-IGZO sensor arrays [77]. These approaches mainly rely on external modules, array-level architectures, or distinguishable response features for perception, classification, and neuromorphic learning. In contrast to these studies, our work focuses on coupled-stimulus decoupling and independent signal reconstruction within a single sensing channel. To determine the representational capacity required for accurate decoupling, we benchmarked several regression families with distinct modeling characteristics, including linear regression (LR), polynomial regression (PR), support vector regression (SVR), gradient-boosted decision trees (XGBoost), and an MLP (Fig. 5a) [77–79]. The corresponding architectures and mathematical formulations are detailed in Fig. S20 and Note S3. The coefficient of determination (*R*^2^) in Fig. 5b indicates that architectures beyond linear regression achieve higher predictive accuracy, suggesting that the mapping between electrical fingerprints and physical stimuli involves higher-order dependencies within this dataset. Among the candidates, the MLP yields the highest R^2^ for both light intensity and temperature, a result further supported by the minimal mean absolute error (MAE) shown in Fig. 5c. This consistent performance is corroborated by the final loss values summarized in Fig. S21 and the minimized mean squared error (MSE) shown in Fig. S22. Based on these evaluation results, a compact three-layer MLP (128–64–32 neurons) was implemented for final decoupling (Fig. 5d). The training loss rapidly converges within the first few hundred epochs (Fig. 5e), while the *R*^2^ during optimization consistently approaches unity (Fig. 5f). To visually compare the predictive behaviors across models, the parity plots for light intensity (0–5 mW/mm^-2^) are provided in Fig. S23 for LR, PR, SVR, and XGBoost, with the corresponding analysis for the MLP presented in Fig. 5g. Similarly, the parity plots for temperature (20–80 °C) for the alternative models are detailed in Fig. S24, while the MLP result is shown in Fig. 5h. On the independent test set, the MLP achieves *R*^2^ values of 0.992 and 0.989 for light intensity and temperature prediction, respectively. The narrow and symmetrically centered residual distributions (Fig. 5i) confirm unbiased and precise decoupling. To further evaluate modal decoupling, we first examined the residual trends with respect to the non-target modality, as shown in Fig. S25 [80]. In both cases, the residual distributions were smoothed using locally weighted scatterplot smoothing (LOWESS) to reveal the underlying trend, and the resulting curves remain nearly horizontal with only moderate fluctuations, indicating weak systematic dependence of the prediction errors on variations in the other sensing variable. To further quantify the decoupling performance, cross-sensitivity (${\mathrm{CS}}$), crosstalk error (${\mathrm{CE}}$), and modal orthogonality index (${\mathrm{OI}}$) were evaluated (definitions in Note S5). The values of ${\mathrm{CS}}_{T \leftarrow L}$ and ${\mathrm{CS}}_{L \leftarrow T}$ were 0.11 °C (mW/mm^2^)^−1^ and 0.0028 mW mm^−2^ °C⁻^1^, respectively, while ${\mathrm{CE}}_{T \leftarrow L}$ and ${\mathrm{CE}}_{L \leftarrow T}$ were 0.55 °C and 0.17 mW mm^−2^. In addition, the ${\mathrm{OI}}$ reached 0.94, indicating strong modal separation between the two sensing channels.

### System Integration and Real-World Environmental Monitoring

**Fig. 6:**
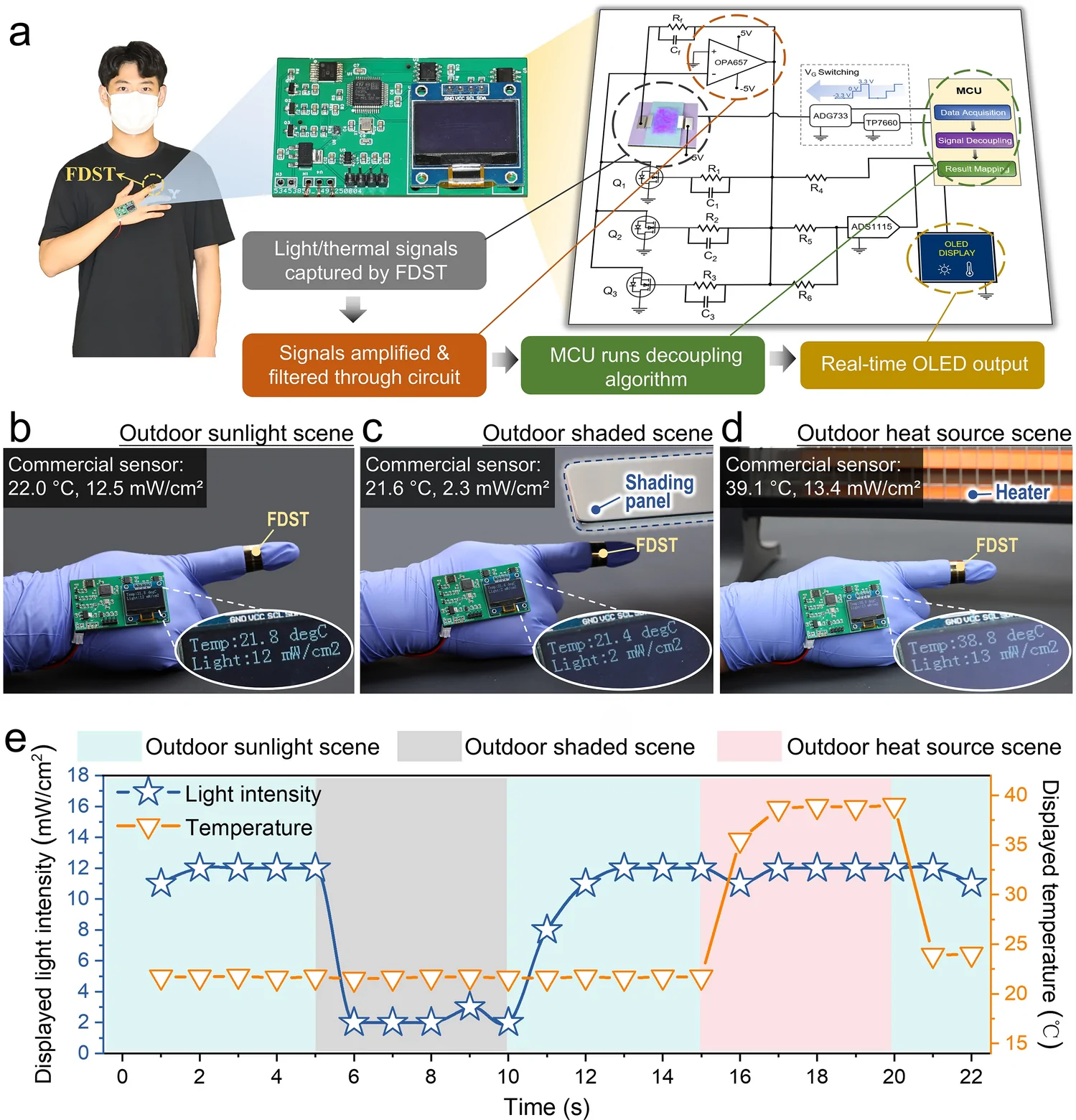
Design, integration, and application of the WDMS. **a** Photograph and system block diagram of the WDMS. **b–d** Real-world outdoor demonstrations of the WDMS under different scenarios: **b** direct sunlight, **c** shaded conditions, and **d** combined photothermal stimulation, benchmarked against commercial sensor references. **e** Time-resolved light intensity and temperature during shadow–recovery and heating–removal cycles

Building upon the high-precision predictions of the MLP-based decoupling model, we developed a wearable sensing system capable of synchronous acquisition, decoupling, and real-time display of ambient light and temperature signals under practical usage conditions (Fig. 6a). To accommodate the wide dynamic range of drain currents spanning several orders of magnitude under different gate biases (*V*_G_ = − 3.3, 0, and + 3.3 V), the system architecture incorporates a three-channel readout circuit with tailored gain settings. This configuration prevents amplifier saturation and ensures high-resolution signal acquisition across all operating regimes. The analog front-end utilizes transimpedance amplifiers (TIA, OPA657) for current-to-voltage conversion, followed by digitization via a multichannel 16-bit ADC (ADS1115). An STM32 microcontroller (MCU) collects the data and directly executes the pretrained MLP model to infer light intensity and temperature without the need for further retraining. Aligning with the intrinsic second-level response time of the FDST to thermal stimuli, the WDMS updates the displayed results at a 1 s interval, which effectively captures the real-time environmental variations while filtering out high-frequency noise.

To rigorously validate the WDMS’s robustness under uncontrolled real-world conditions, we benchmarked our system against commercial sensors across three progressively challenging scenarios: baseline sunlight, shadow occlusion, and compound photothermal stimulation. The light intensity and temperature values were benchmarked against commercial reference instruments, namely a Thorlabs PM100D optical power meter (Thorlabs, USA) and a Fluke 54 II digital thermometer (Fluke Corporation, USA), respectively. Initially, under direct sunlight (Fig. 6b), the system demonstrated high-fidelity tracking, yielding values (21.8 °C, 12 mW cm^−2^) nearly identical to the commercial reference (22.0 °C, 12.5 mW cm^−2^), thereby establishing a solid accuracy baseline. Crucially, the system’s decoupling capability was tested by introducing a shadow (Fig. 6c). While the commercial sensor recorded a sharp illumination drop to 2.3 mW cm^−2^, our system accurately reproduced this transition (2 mW cm^−2^) without inducing any erroneous thermal drift (maintaining a stable output at 21.4 °C). This result highlights the independence of the predicted optical signal from thermal interference. Finally, in the most demanding scenario involving simultaneous intense irradiation and local heating (Fig. 6d), the system successfully resolved a high target temperature (38.8 °C) against a high-light background (13 mW cm^−2^), matching the reference (39.1 °C, 13.4 mW cm^−2^) with minimal error. This confirms that the MLP-based model achieves excellent modal orthogonality, effectively eliminating crosstalk even within a wide dynamic range of compound environmental stimuli.

To further evaluate the system’s robustness under continuous dynamic conditions, we recorded a real-time demonstration covering complete “shadow–recovery” and “heating–removal” cycles (Video S1), with corresponding temporal data presented in Fig. 6e. Throughout the dynamic testing, the system exhibits fast transient response, accurately tracking abrupt optical occlusion and temperature rise events with negligible hysteresis during the subsequent recovery phases. Importantly, the sensing platform maintains strict modal independence: pronounced variations in one environmental parameter produce no measurable perturbation in the other. These results confirm that the system achieves real-time, crosstalk-free decoupling even under compound, time-varying environmental stimuli.

## Conclusions

In this work, we developed a flexible dual-modal sensing transistor (FDST) capable of simultaneously detecting light intensity and temperature within a single sensing unit. By integrating electrospun ZnO nanofibers with an IGZO thin-film transistor, the device leverages defect-mediated optical excitation and thermally activated interfacial potential modulation to generate strong photothermal responses. As a result, the FDST achieves a broadband responsivity of up to 2.69 A W^−1^ and a temperature coefficient of 0.071 °C^−1^, demonstrating reliable sensitivity to both optical and thermal stimuli. To resolve the intrinsic coupling between these signals, a multibias electrical readout strategy was introduced to encode the stimuli into a bias-dependent current fingerprint. A lightweight MLP was then employed to decode these electrical features, enabling accurate reconstruction of light intensity and temperature with coefficients of determination of 0.992 and 0.989, respectively. Importantly, the FDST exhibits excellent mechanical robustness, maintaining stable sensing performance even after 10,000 bending cycles at a bending radius of 2 mm, highlighting its suitability for flexible and wearable electronics. Furthermore, the device was integrated into a wearable dual-modal monitoring system that enables real-time environmental sensing with negligible modal cross-interference under outdoor conditions. Overall, this work demonstrates a compact device-algorithm co-design strategy for multimodal sensing, providing a scalable pathway toward intelligent wearable perception systems and next-generation electronic skins.

## Supplementary Information

Below is the link to the electronic supplementary material.

Supplementary file1 (DOCX 5353 KB)

Supplementary file2 (MP4 26496 KB)
