# Supplementary material for: Flexible Dual-Modal Sensing Transistor Enabled by Deep Learning Decoupling for Independent Light and Temperature Reconstruction
Source: Nanomicro Lett. 2026 Jul 7;18:429. doi: 10.1007/s40820-026-02285-7 (PMC13342015; doi:10.1007/s40820-026-02285-7)
Supplement: Supplementary file 1 — Supplementary file1 (DOCX 5353 KB) [file 40820_2026_2285_MOESM1_ESM.docx]

Supporting Information for

**Flexible Dual-Modal Sensing Transistor Enabled by Deep Learning Decoupling for Independent Light and Temperature Reconstruction**

Shilin Lu^1^, Ji Hoon Han^1^, Dong Keun Lee^2^, Sun Min Song^1^, Huixin Yu^3^, Sujin Jung^1^, Lu Zhang^1^, Zhao Yao^3^, Jong Bin An^1, 4*^ and Hyun Jae Kim^1*^

^1^School of Electrical and Electronic Engineering, Yonsei University, Seoul 03722, Republic of Korea

^2^Department of Integrated Display Engineering, Yonsei University, Seoul 03722, Republic of Korea

^3^College of Electronic and Information, Qingdao University, Qingdao 266071, China

^4^BIT Micro Fab Research Center, Yonsei University, Seoul 03722, Republic of Korea

*Corresponding authors. E-mail: [jongbin1996@yonsei.ac.kr](mailto:jongbin1996@yonsei.ac.kr) (Jong Bin An); [hjk3@yonsei.ac.kr](mailto:hjk3@yonsei.ac.kr) (Hyun Jae Kim)

**Note S1 Evaluation metrics used to determine the optimal electrospinning deposition time**

To determine the optimal electrospinning deposition time for the ZnO NFs, the device responses under optical and thermal stimuli were quantitatively evaluated using internal metrics based on drain current variation ($S_{I_{on}}$) and threshold voltage shift ($S_{V_{th}}$), defined as follows:

$S_{I_{on},light}=\frac{\Delta I}{I_{0}\cdot\Delta P}$ (S1)

$S_{I_{on},temp}=\frac{\Delta I}{I_{0}\cdot\Delta T}$ (S2)

$S_{V_{th},light}=\frac{\Delta V_{th}}{I_{0}\cdot\Delta P}$ (S3)

$S_{V_{th},temp}=\frac{\Delta V_{th}}{I_{0}\cdot\Delta T}$ (S4)

where $I_{0}$ represents the initial current, $\Delta P$ is the variation in light density, and $\Delta T$ is the temperature change. Both optical- and thermal-response-related metrics exhibit a non-monotonic dependence on the electrospinning time, reaching a maximum at 40 s (**Fig. S5**). This behavior reflects a critical balance in the nanofiber network density: while a moderate density provides abundant sensing interfaces, excessive accumulation may enhance light scattering or cause mutual shielding of interfacial states. Consequently, a deposition time of 40 s was adopted for all subsequent device characterizations.

**Note S2 Mathematical models used for surface charge modulation in FEA simulation**

To quantify the modulation effects of light and temperature on the ZnO surface band structure, this study employs literature-supported independent models to describe the variations in net surface charge density (σ).

For the light-induced effect, the sub-gap states introduced by oxygen vacancies and surface defects in ZnO facilitate the generation of photocarriers under visible light. The resulting defect filling and photogating effect are approximated as a saturation function of light intensity (P) expressed as:

$\Delta\sigma_{light}= k_{L}\cdot\left( 1 - e^{-\frac{P}{P_{0}}} \right)$ (S5)

This formulation aligns with the persistent photoconductivity and photogating mechanisms reported in ZnO nanostructures [S1, S2].

For the temperature-induced effect, the adsorption–desorption processes of surface oxygen species and hydroxyl groups follow Arrhenius kinetics. The resulting variation in surface charge is approximated by the following exponential relationship:

$\Delta\sigma_{heat}= k_{T}\cdot\left( e^{-\frac{E_{des}}{k_{B}T}}- e^{-\frac{E_{des}}{k_{B}T_{0}}} \right)$ (S6)

where *T_0_* represents the initial temperature. This model is consistent with the chemical kinetics of gas-surface interactions in metal oxide sensors [S3, S4].

**Note S3 Mathematical Formulation of the Regression Models**

To ensure reproducibility and clarity, we summarize the mathematical formulations of the regression models benchmarked in this work. The three-bias electrical fingerprint vector *x* is defined as:

$x = \left[ I_{D,-3.3V}, I_{D,0V}, I_{D,+3.3V} \right]^{T}\in\mathbb{R}^{3}$ (S7)

The objective is to learn a mapping function $f : \mathbb{R}^{3}\to\mathbb{R}^{2}$ to predict the output vector $y = \left( L, T \right)^{T}$, where *L* and *T* represent light intensity and temperature, respectively.

1. LR

LR assumes a linear mapping between input features and outputs, expressed as:

$ŷ = W x + b$ (S8)

where $W \in\mathbb{R}^{2\times3}$ and $b \in\mathbb{R}^{2}$ are the weight matrix and bias vector, respectively.

(2) PR

PR extends the linear model by introducing higher-order feature interactions. The augmented feature vector $\Phi\left( x \right)$ is defined as:

$\Phi\left( x \right)= \left( x_{1}, x_{2}, x_{3}, x_{1}^{2}, x_{2}^{2}, x_{3}^{2}, x_{1}x_{2}, x_{1}x_{3}, x_{2}x_{3} \right)^{T}$ (S9)

The prediction is given by $ŷ = W \Phi\left( x \right)+ b$.

(3) SVR

For multi-target prediction, independent SVR models were trained for each stimulus using the following function form:

$f\left( x \right)= \sum_{i=1}^{N} \alpha_{i}K\left( x, x_{i} \right)+ b$ (S10)

A radial basis function (RBF) kernel was employed to handle nonlinearities, expressed as:

$K\left( x, x_{i} \right)=\exp\left( -\gamma\left\| x - x_{i} \right\|^{2} \right)$ (S11)

(4) XGBoost

XGBoost implements an additive ensemble of *M* regression trees, expressed as:

$ŷ = \sum_{m=1}^{M} g_{m\left( x \right)}$ (S12)

where each $g_{m}$ represents an individual regression tree optimized via gradient boosting with structural regularization.

(5) MLP

The MLP implements a nonlinear mapping through stacked fully connected layers. The hidden-layer transformations and final output are expressed as:

$h_{n}= \sigma\left( W_{n}h_{n-1}+ b_{n} \right)$ (S13)

$ŷ = W_{out}h_{last}+ b_{out}$ (S14)

where $\sigma$ (·) denotes the Rectified Linear Unit (ReLU) activation function.

All models were implemented using the PyTorch framework with identical train–test splits and optimization protocols to ensure a fair comparison of their predictive capacities.

**Note S4 Physical Basis and Optimization of Multi-Bias Selection**

The three gate biases V_G_ = −3.3 V, 0 V, and +3.3 V were selected based on both device physics and system-level considerations. These biases sample distinct regions of the transfer characteristics with markedly different sensitivities to light-induced defect excitation and thermally activated surface processes. In addition, the ± 3.3 V range matches the supply voltage of the microcontroller unit in the wearable platform, enabling direct implementation without additional voltage conversion and minimizing power consumption.

To assess the effectiveness of this selection, a retrospective ablation study was performed using the full transfer curves measured by a Keithley 4200A. Drain currents at different bias combinations were extracted and evaluated using multilayer perceptron (MLP) models with a shared architectural template: the input dimension varies with the number of bias points, while the hidden layers (128–64–32) and training protocol are fixed. This controls model capacity and ensures that performance differences primarily reflect input feature dimensionality.

As summarized in **Table S6**, single-bias inputs show limited predictive accuracy, while dual-bias combinations substantially improve both R^2^ and MAE. The three-bias configuration achieves the best performance across both light intensity and temperature prediction. The results support the use of the three-bias configuration as a practical choice, taking into account both decoupling performance and system-level considerations such as circuit complexity and power consumption.

**Note S5 Quantitative Evaluation of Modal Decoupling**

To isolate cross-interference effects, the dataset was grouped into subsets with discretized temperature values for evaluating the influence of light intensity, and subsets with discretized light-intensity values for evaluating the influence of temperature. Within each subset, only the non-target modality varies, while the target variable remains effectively constant. The prediction residuals are defined as:

$\varepsilon_{T,i}=T_{pred,i}-T_{true,i}$ (S15)

$\varepsilon_{L,i}=L_{pred,i}-L_{true,i}$ (S16)

1. **Cross-sensitivity**

The cross-sensitivity of temperature prediction with respect to light-intensity variation ($CS_{T\leftarrow L}$) is defined as:

$CS_{T\leftarrow L}=\frac{1}{K_{T}}\sum_{k=1}^{K_{T}} \left| \frac{d\varepsilon_{T}^{\left( k \right)}}{dL} \right|$ (S17)

Similarly, the cross-sensitivity of light-intensity prediction with respect to temperature variation ($CS_{L\leftarrow T}$) is defined as:

$CS_{L\leftarrow T}=\frac{1}{K_{L}}\sum_{k=1}^{K_{L}} \left| \frac{d\varepsilon_{L}^{\left( k \right)}}{dT} \right|$ (S18)

where $K_{T}$ and $K_{L}$denote the number of subsets under fixed-temperature and fixed-light-intensity conditions, respectively. $\varepsilon_{T}^{\left( k \right)}$ and $\varepsilon_{L}^{\left( k \right)}$ represents the residual function within the $k$-th subset. The derivatives are numerically estimated via linear fitting within each subset and quantify the residual dependence of the prediction error on the non-target modality.

1. **Crosstalk error**

The crosstalk error of temperature prediction with respect to light-intensity variation ($CE_{T\leftarrow L}$) is defined as:

$CE_{T\leftarrow L}=CS_{T\leftarrow L}\cdot\Delta L$ (S19)

The crosstalk error of temperature prediction with respect to light-intensity variation ($CE_{L\leftarrow T}$) is defined as:

$CE_{L\leftarrow T}=CS_{L\leftarrow T}\cdot\Delta T$ (S20)

where $\Delta L=L_{\max}-L_{\min}$ and $\Delta T=T_{\max}-T_{\min}$ denote the tested ranges of light intensity and temperature, respectively.

1. **Modal orthogonality index**

The modal orthogonality index ($OI$) is defined as:

$OI=1-\left| \rho\left( \varepsilon_{L},\varepsilon_{T} \right) \right|$ (S21)

where $\varepsilon_{L}=\left( \varepsilon_{L,1},\varepsilon_{L,2},\ldots,\varepsilon_{L,N} \right)$ and $\varepsilon_{T}=\left( \varepsilon_{T,1},\varepsilon_{T,2},\ldots,\varepsilon_{T,N} \right)$ denote the residual vectors.

**Supplementary Figures and Tables**

**
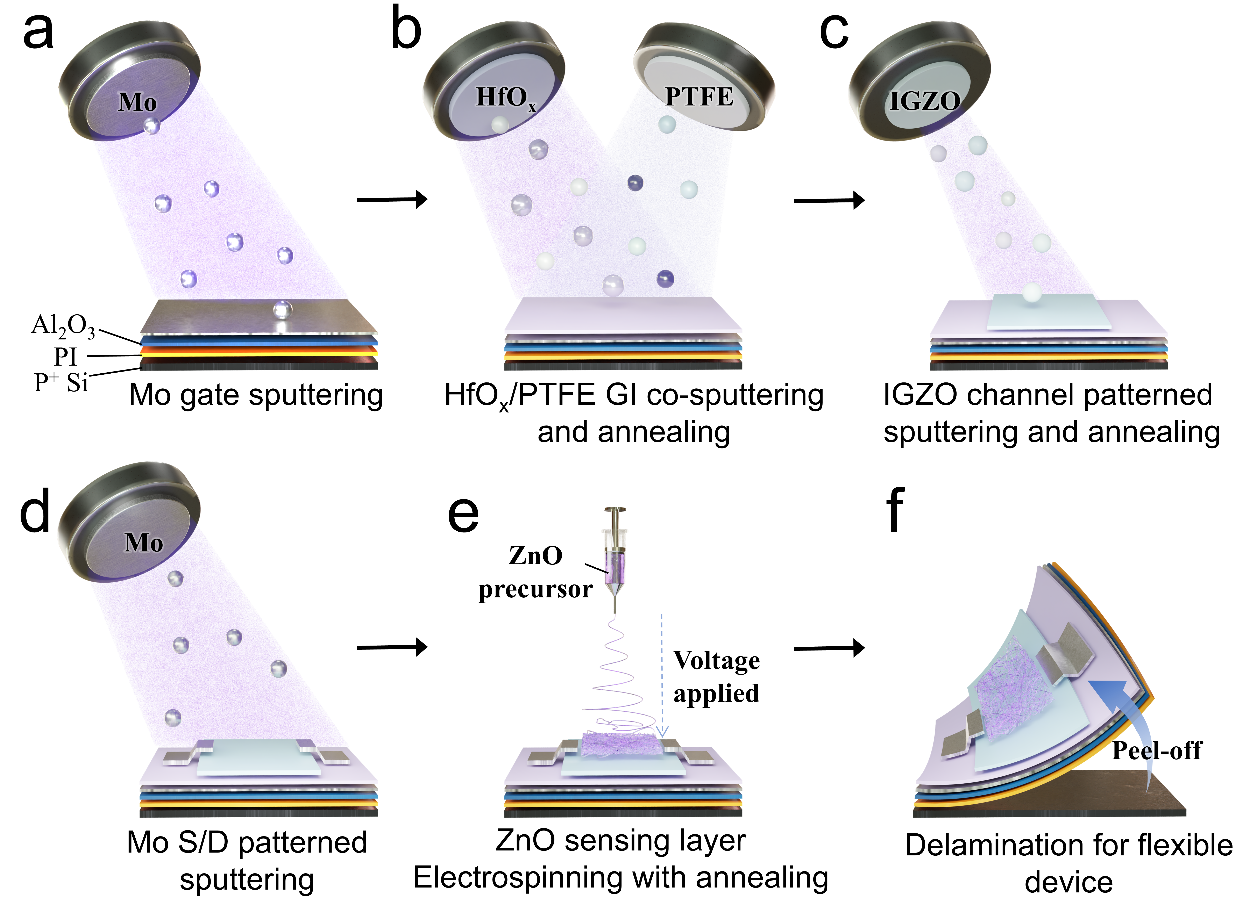
**

**Fig. S1** Schematic illustration of the step-by-step fabrication process for the FDST. The workflow is initiated by **a** the deposition of the Mo bottom gate electrode onto the Al_2_O_x_-buffered PI substrate via RF sputtering. Subsequently, **b** the HfO_x_/PTFE hybrid GI is formed by RF co-sputtering, followed by thermal annealing. **c** The IGZO channel layer is deposited via sputtering through a shadow mask and annealed to activate semiconductor properties. **d** Mo source and drain electrodes are patterned to define the channel dimensions. **e** The ZnO nanofiber sensing network is constructed via electrospinning of the precursor solution and subsequent annealing. To conclude the process, **f** the flexible device is obtained via delamination from the P^+^ Si substrate.


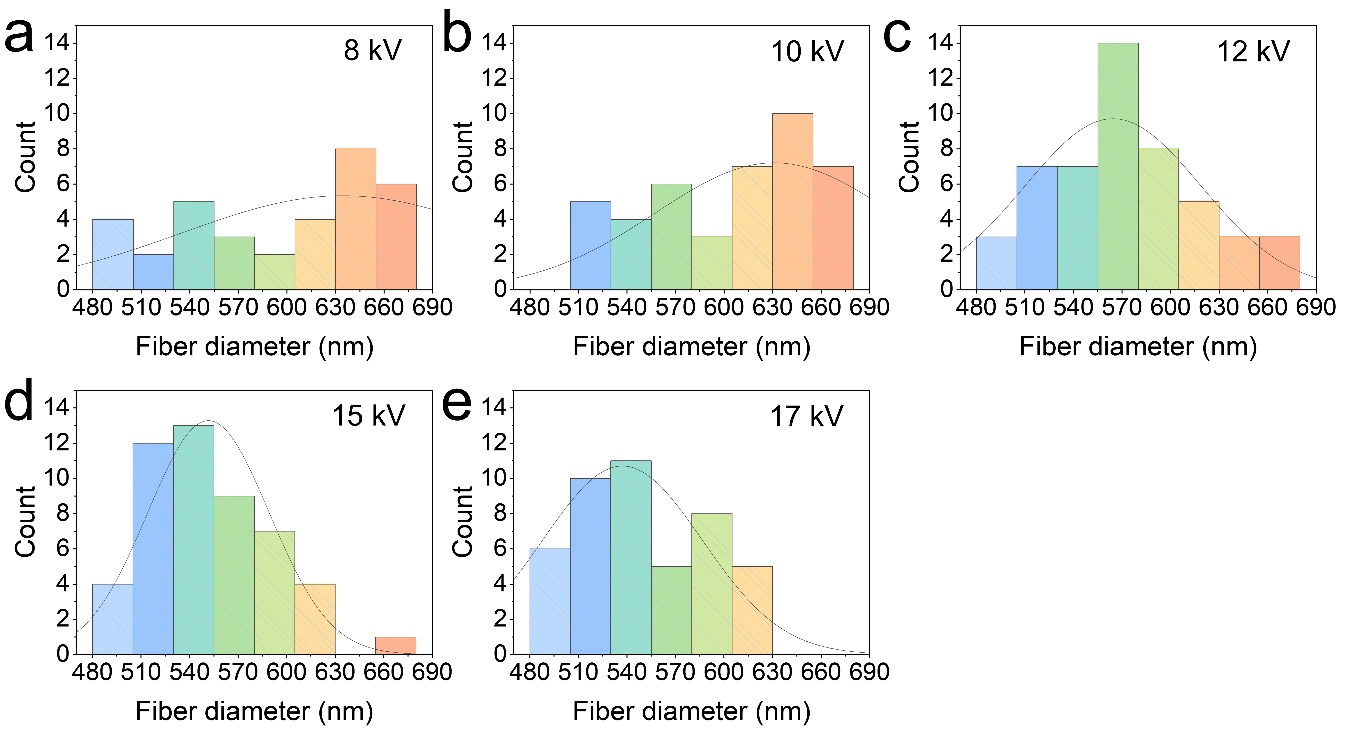


**Fig. S2** Diameter distribution histograms of ZnO NFs electrospun under different applied voltages.

**
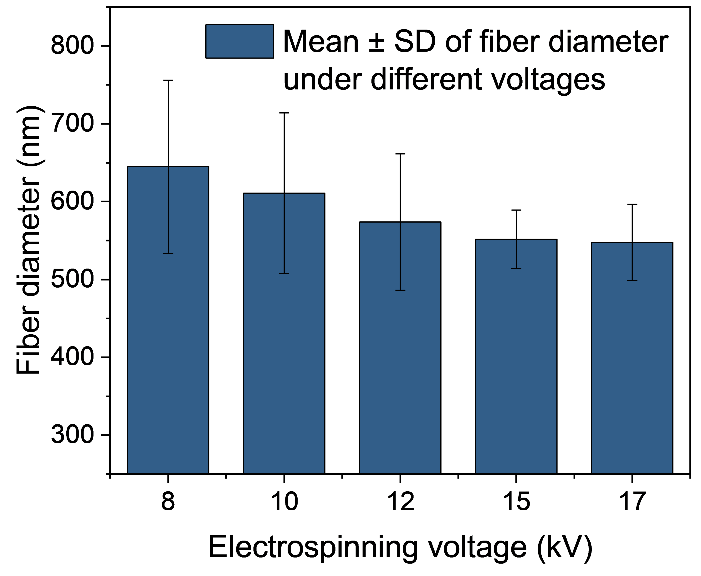
**

**Fig. S3** Statistical analysis of the average diameter and SD of ZnO NFs fabricated under different electrospinning voltages (8–17 kV).


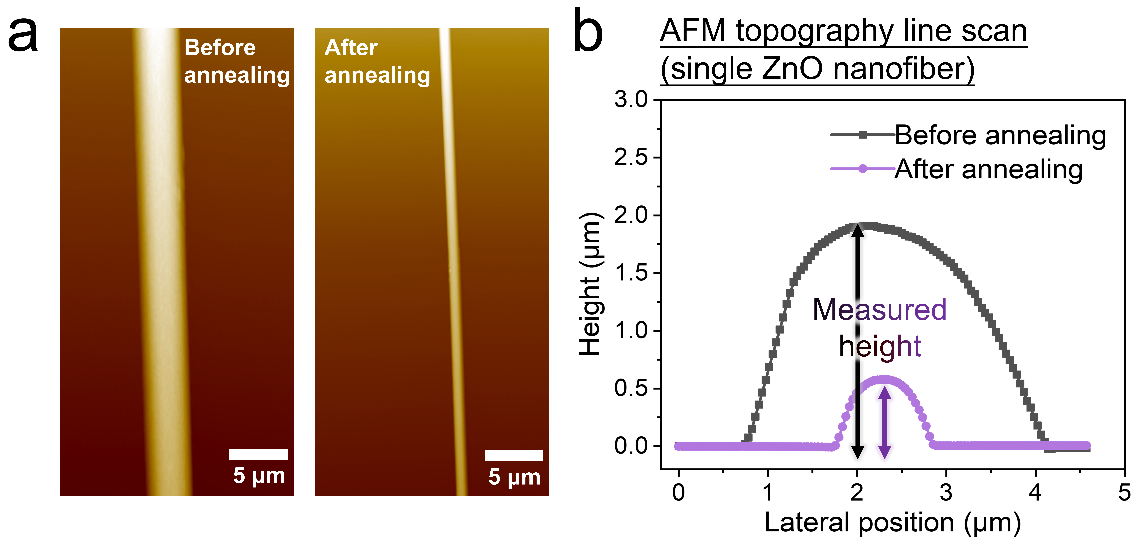


**Fig. S4** AFM characterization of a single ZnO nanofiber before and after thermal annealing. (a) Topography images and (b) corresponding height profiles of the same nanofiber. The RMS roughness is 129.25 nm before annealing and 131.16 nm after annealing.


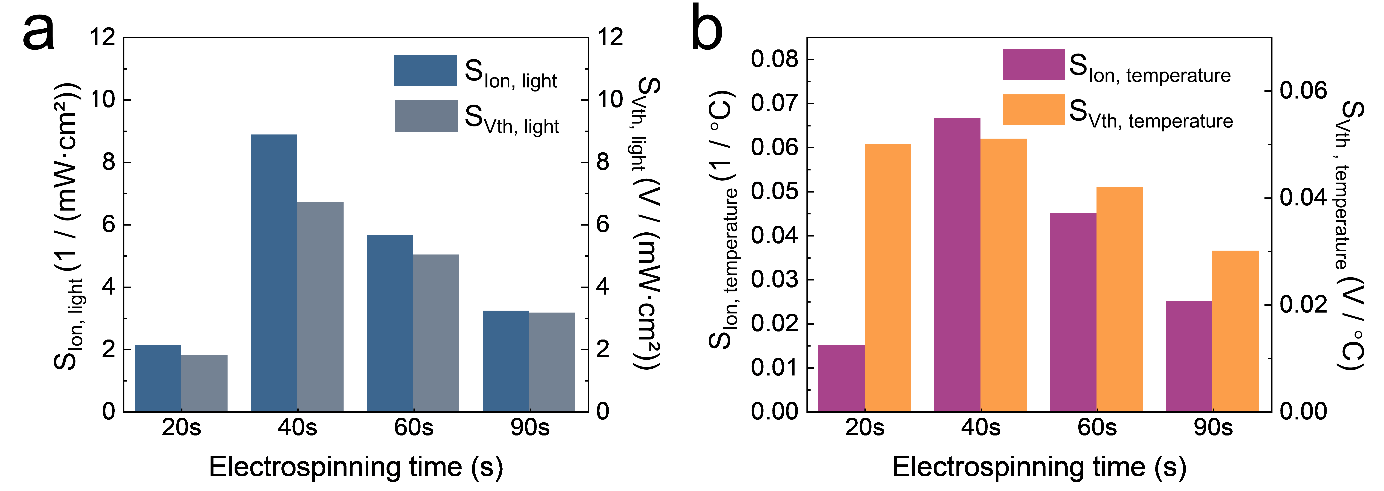


**Fig. S5** Determination of the optimal electrospinning deposition time for ZnO NFs. (a) Light-response-related and (b) temperature-response-related metrics, extracted using the sensitivity definitions in **Note S1** (**Supplementary Information)**, plotted as a function of electrospinning time (20 s, 40 s, 60 s, and 90 s).


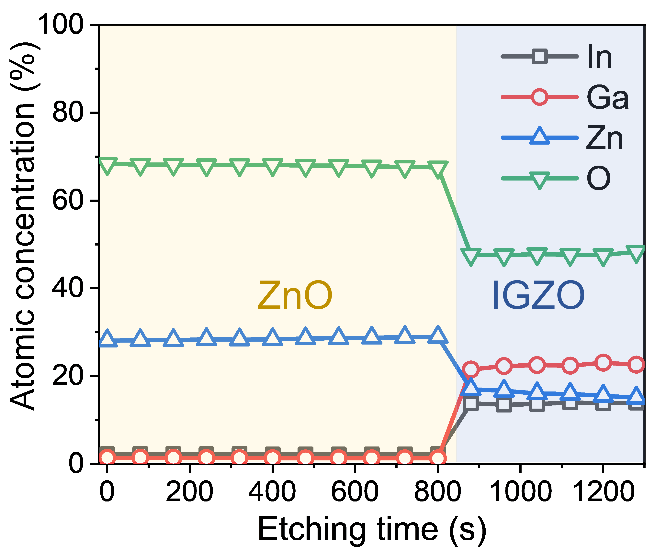


**Fig. S6** Atomic concentration depth profiles of the ZnO/IGZO heterojunction.


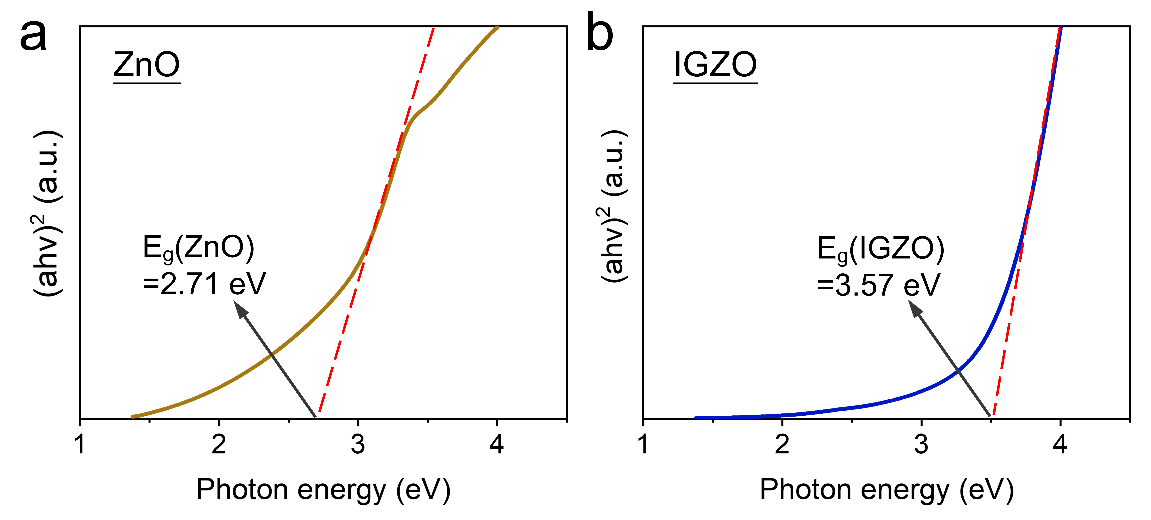


**Fig. S7** Tauc plot of (αhv)² as a function of photon energy for **a** ZnO and **b** IGZO.


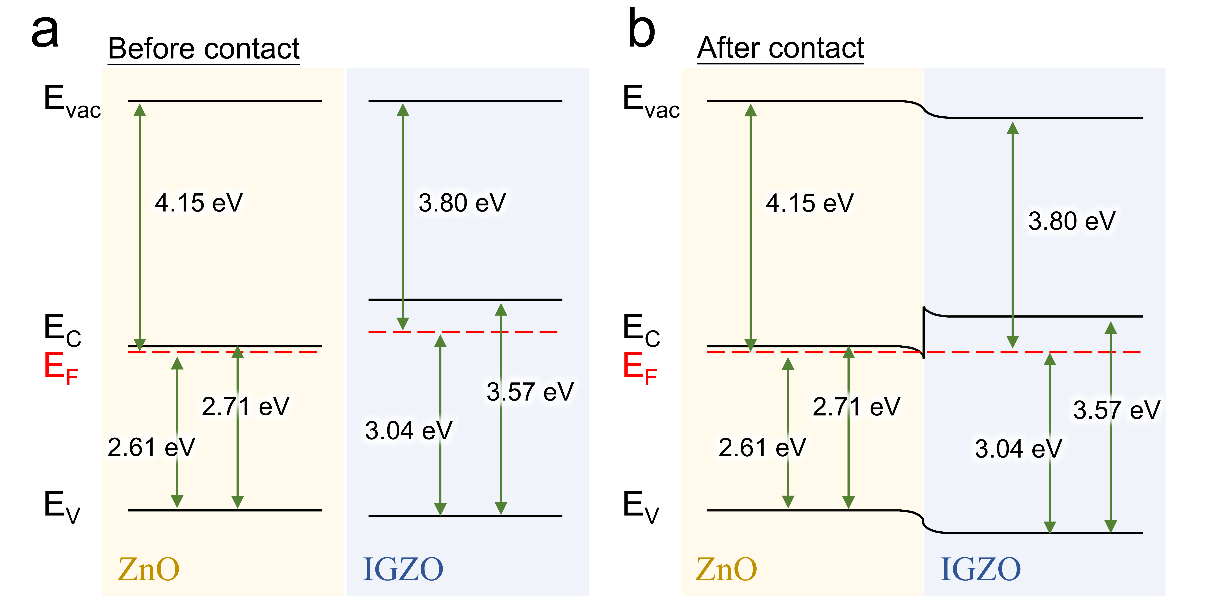


**Fig. S8 a** Energy band diagrams of ZnO and IGZO before contact. **b** Energy band alignment of the ZnO/IGZO heterojunction after contact.


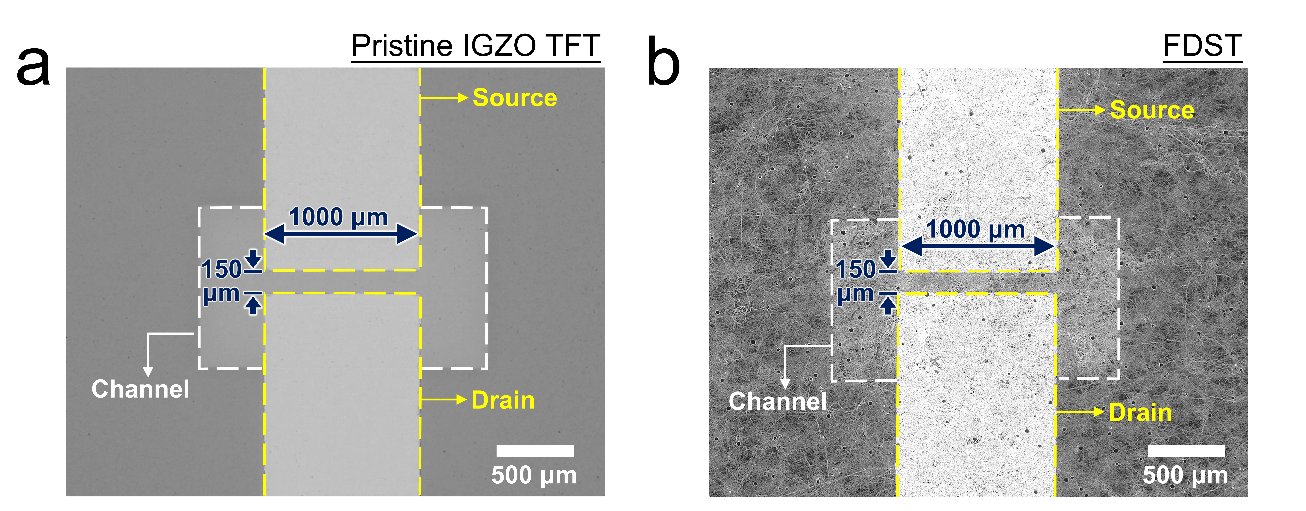


**Fig. S9 a** Top-view optical micrograph of the pristine IGZO TFT. **b** Top-view optical micrograph of the fabricated FDST.


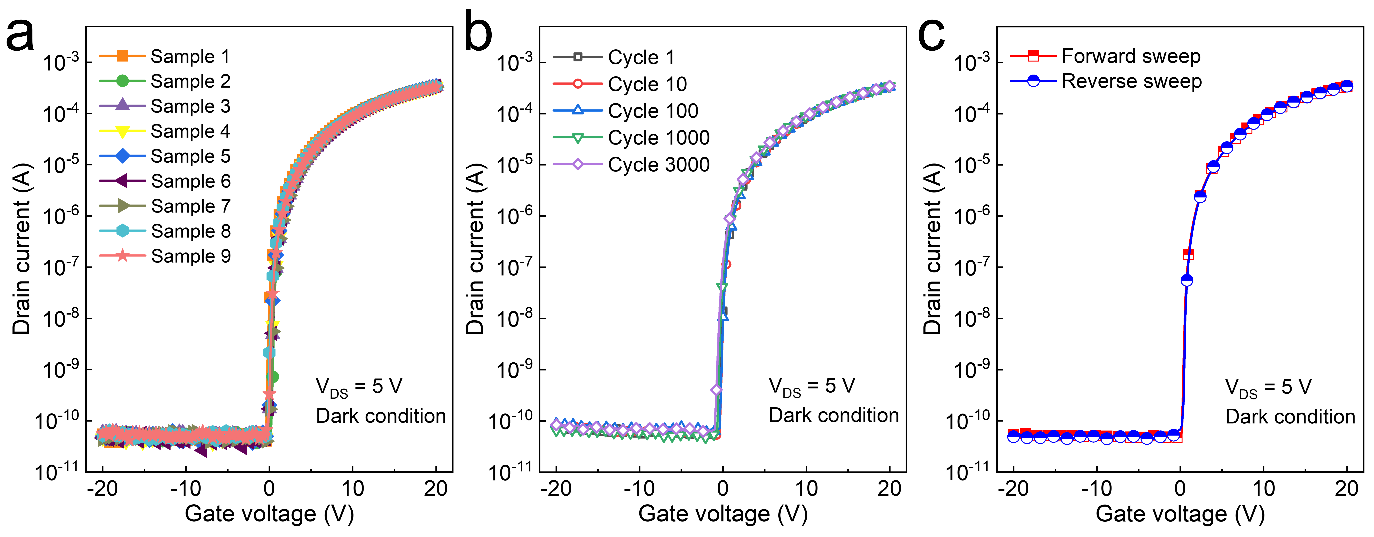


**Fig. S10** Electrical characterization of the FDST. **a** Transfer curves of nine independent devices measured under dark conditions. **b** Transfer curves measured during cyclic operation up to 3000 cycles under dark conditions. **c** Transfer curves obtained from forward and reverse gate-voltage sweeps under dark conditions.


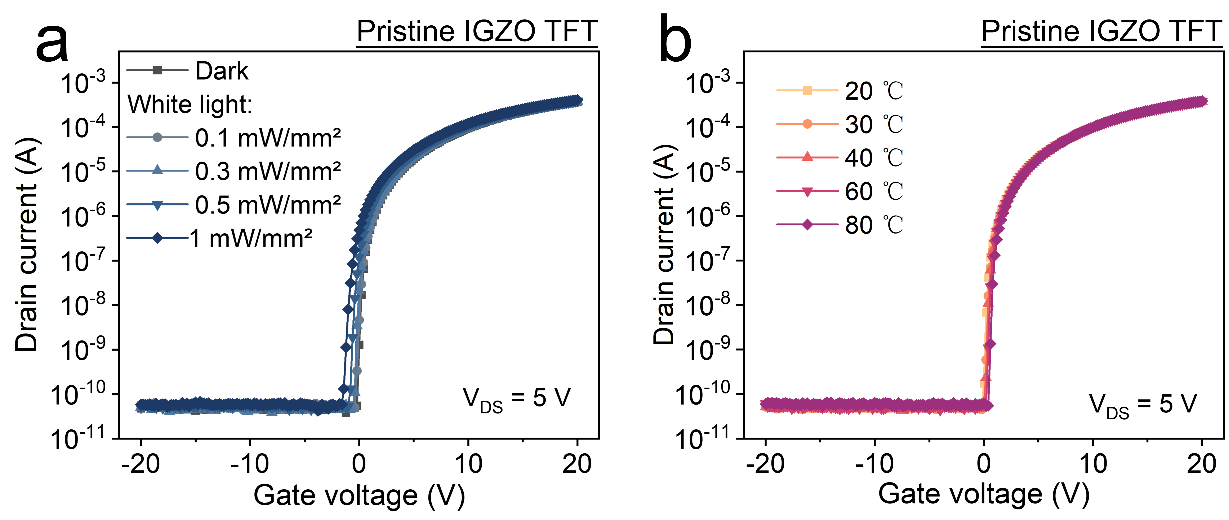


**Fig. S11** **a** Transfer characteristics of the pristine IGZO TFT measured under dark conditions and under white light illumination with different intensities (0.1, 0.3, 0.5, and 1 mW/mm²). **b** Transfer characteristics of the pristine IGZO TFT measured at different temperatures (20, 30, 40, 60, and 80 ℃) in the dark.


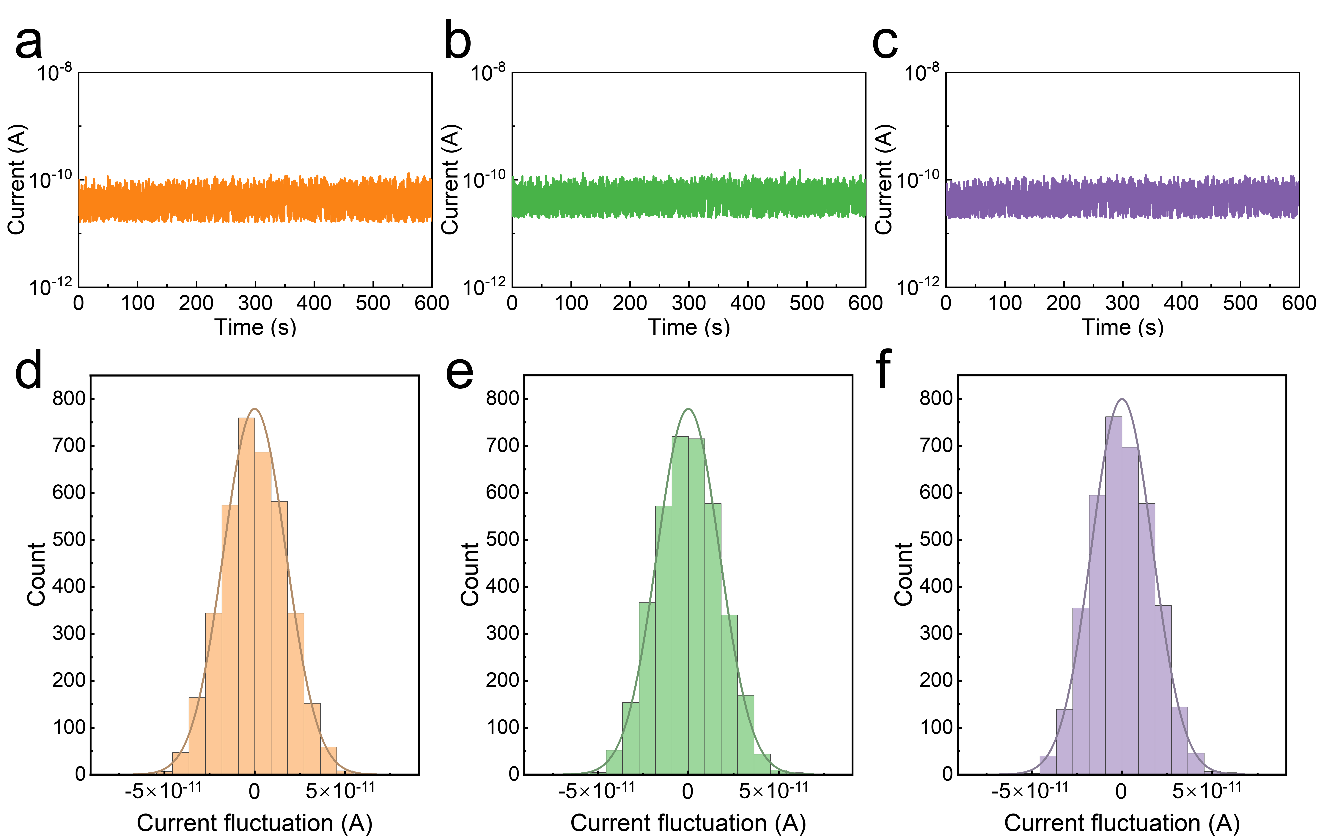


**Fig. S12** Time-domain dark-current fluctuation and statistical analysis under a representative operating condition. **a–c** Drain current versus time for three independent measurements, showing stable fluctuations after the initial transient. **d–f** Corresponding histograms of current fluctuation extracted from the steady-state region, together with Gaussian fits, confirming stationary noise behavior and enabling reliable extraction of the RMS noise current.


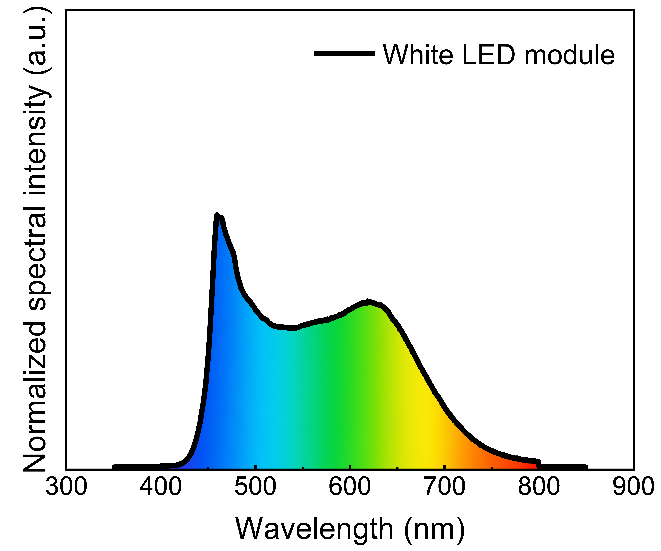


**Fig. S13** Spectral distribution of the white LED.


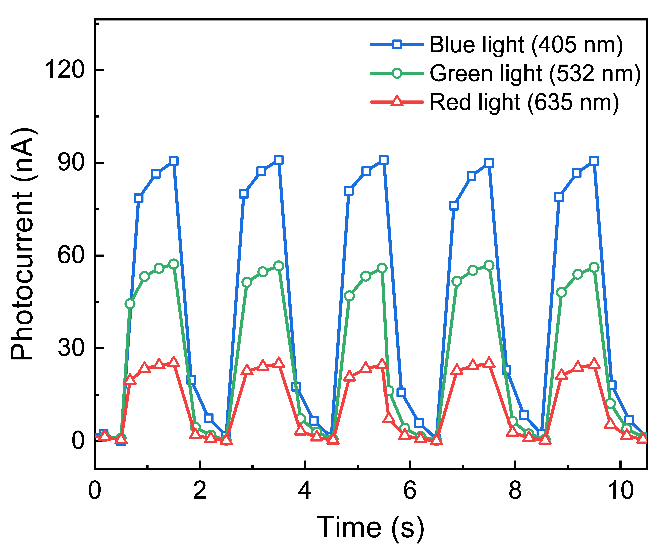


**Fig. S14** Transient photocurrent responses of the FDST under monochromatic illumination at 405, 532, and 635 nm at a light intensity of 1 mW/cm^2^.


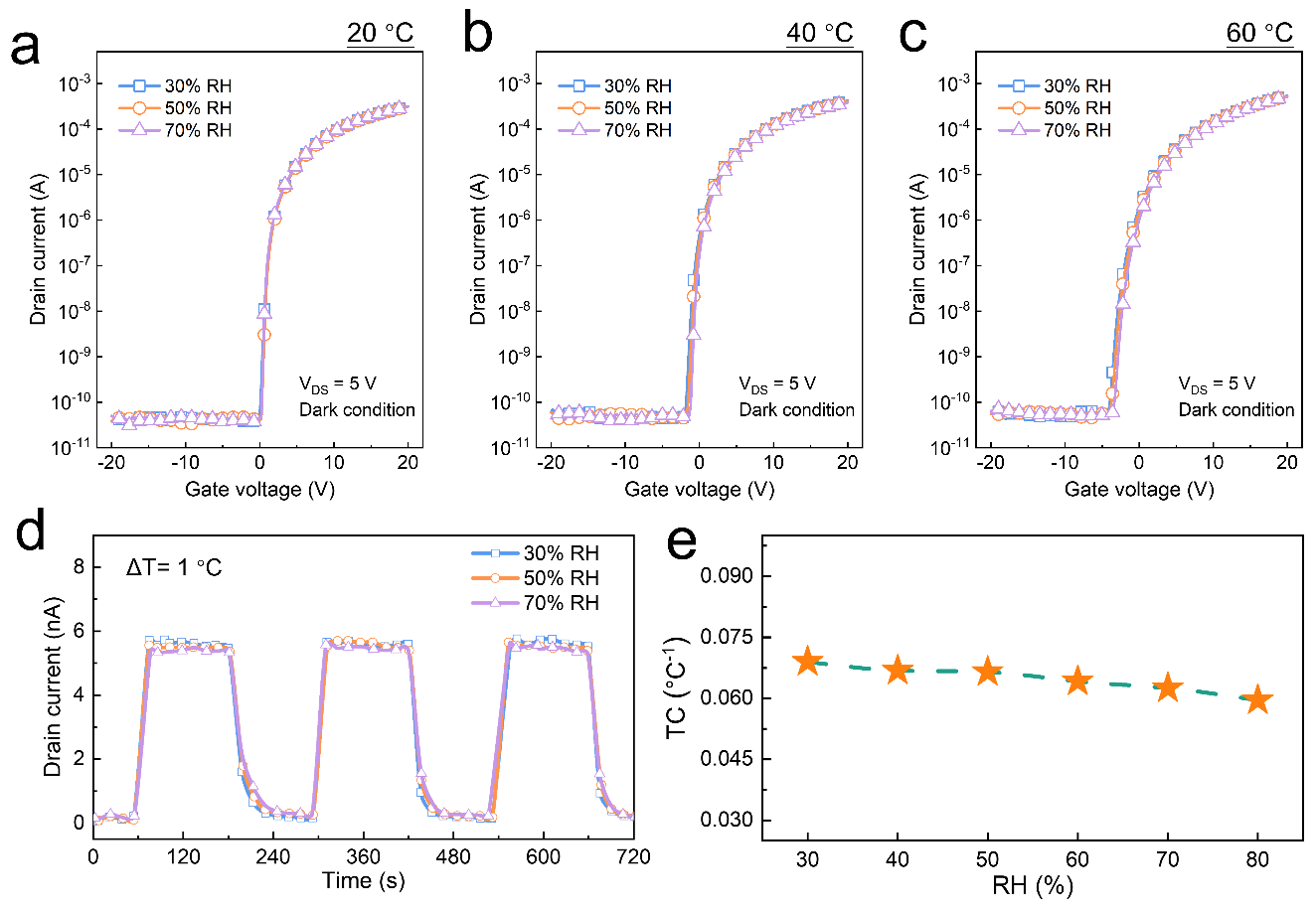


**Fig. S15** Humidity-dependent thermal sensing characteristics of the FDST. **a–c** Transfer characteristics measured at 20, 40, and 60 ℃ under different RH levels of 30%, 50%, and 70%. **d** Dynamic drain-current responses under repeated temperature cycling at different RH levels. **e** Extracted $TC$ as a function of RH.


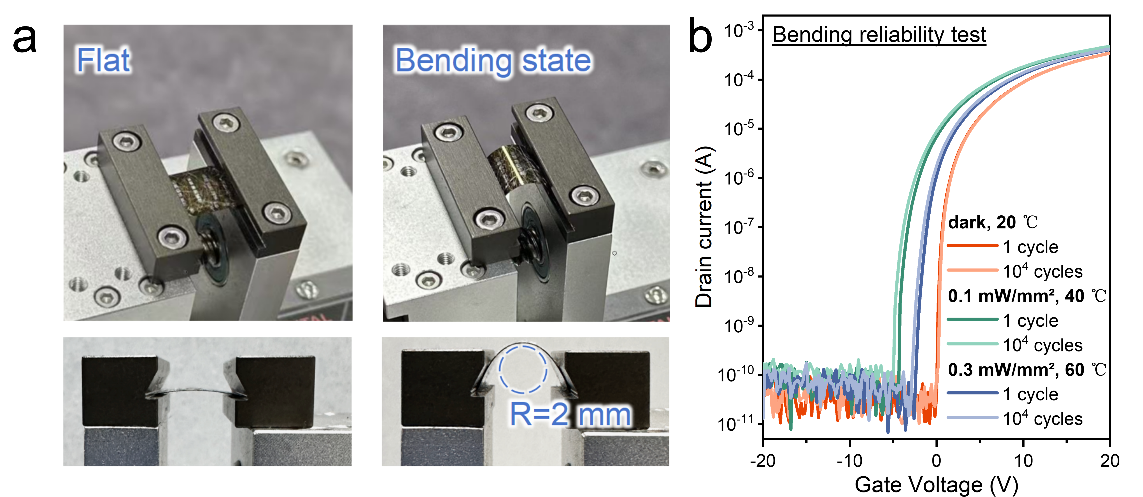


**Fig. S16 a** Photographs of the FDST mounted on a customized bending platform in the flat state and under bending, with optical images showing the bending configuration and radius. **b** Transfer characteristics measured before and after repeated bending cycles at a bending radius of 2 mm under different combined optical and thermal conditions.


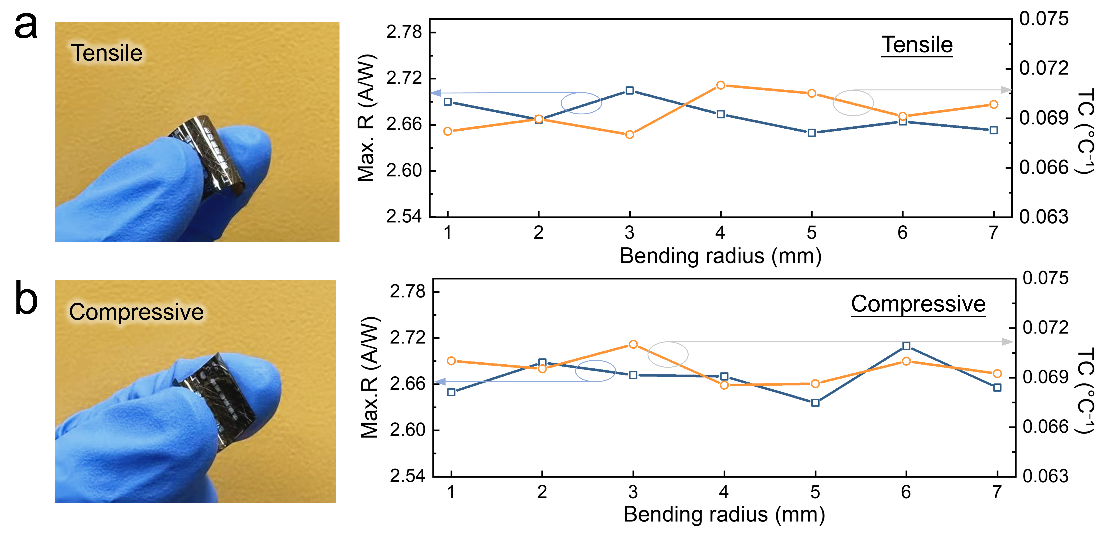


**Fig. S17** Mechanical bending stability of the FDST under **a** tensile and **b** compressive deformation. Left: optical images of the device under bending. Right: variation of Max. $R$ and $TC$ as functions of bending radius.


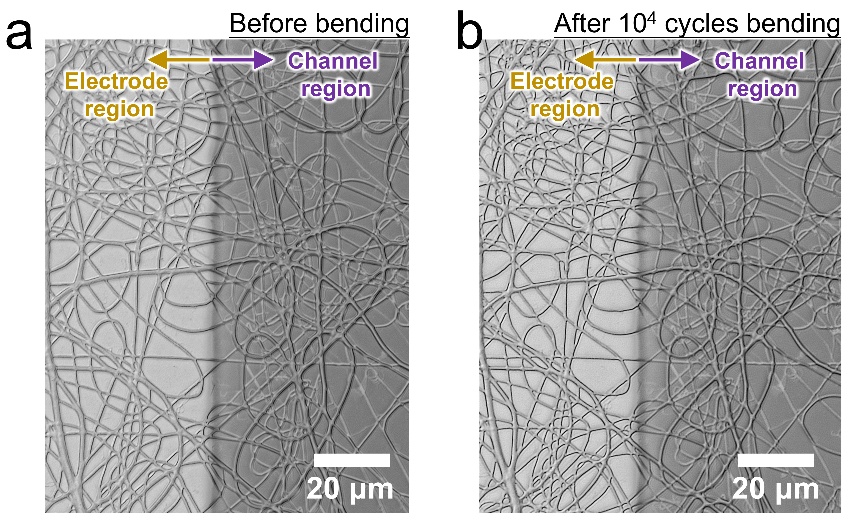


**Fig. S18** Enlarged optical micrographs of the device region: **a** before bending and **b** after 10,000 bending cycles.


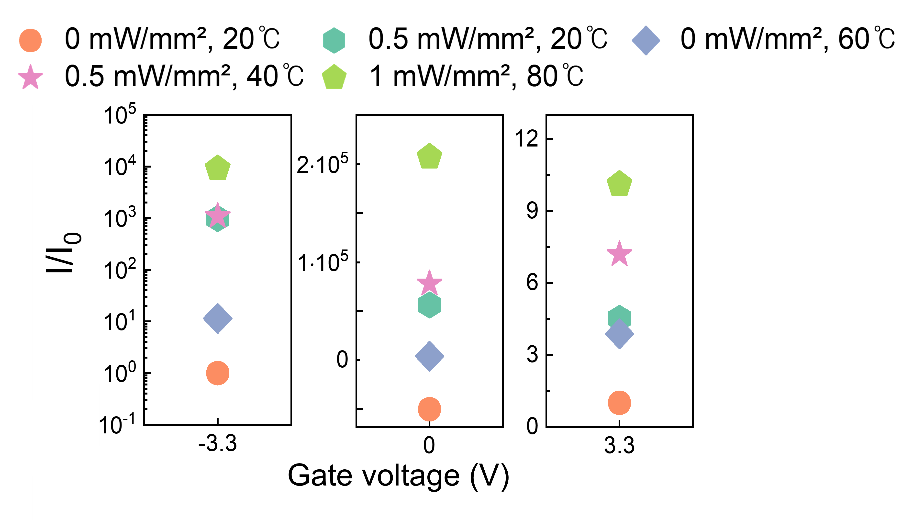


**Fig. S19** Bias-dependent drain-current response of the FDST under representative combinations of light intensity and temperature at V_G_ of -3.3, 0, and +3.3 V.


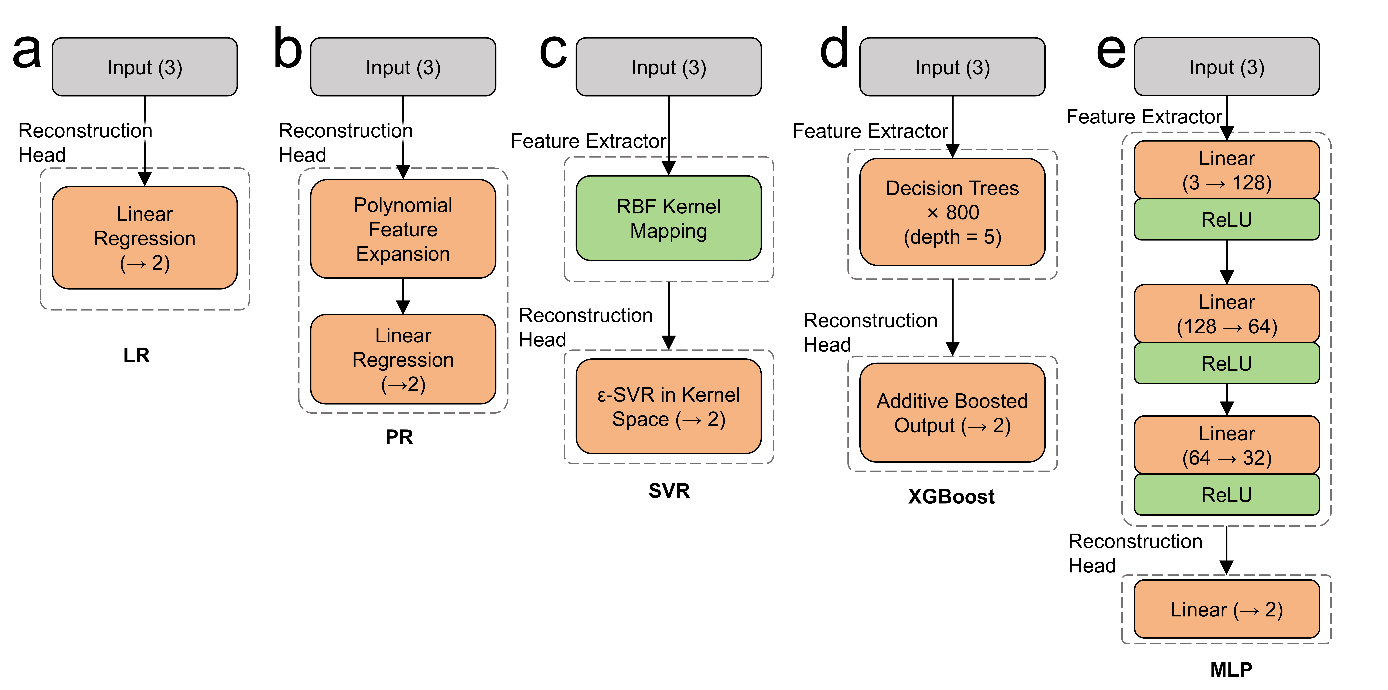


**Fig. S20** Schematic architectures of the regression models: **a** LR, **b** PR, **c** SVR, **d** XGBoost, and **e** MLP.


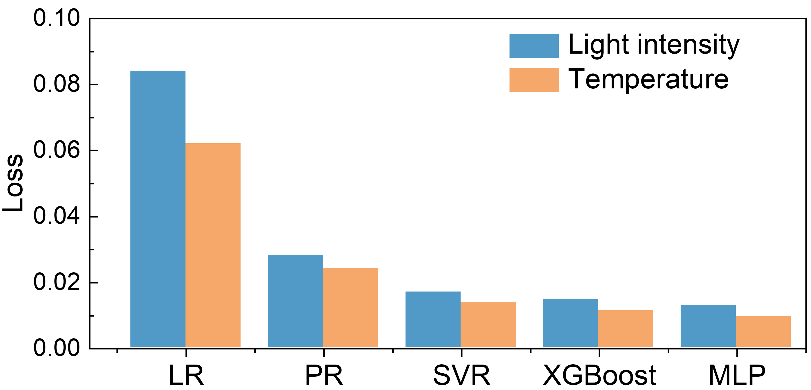


**Fig. S21** Loss for light intensity and temperature prediction across models.


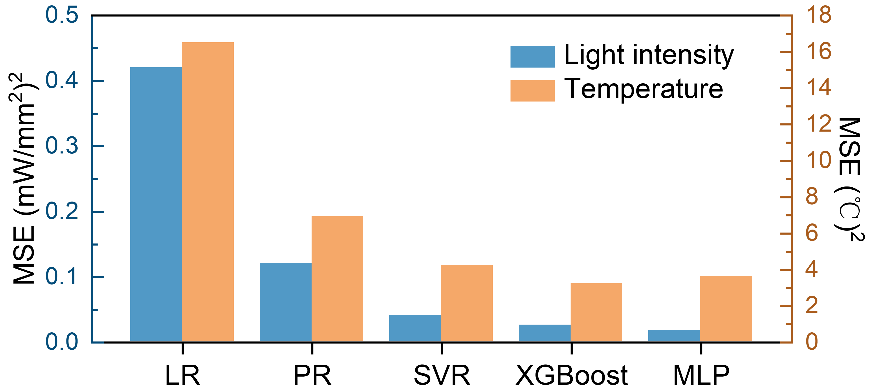


**Fig. S22** MSE for light intensity and temperature prediction across models.


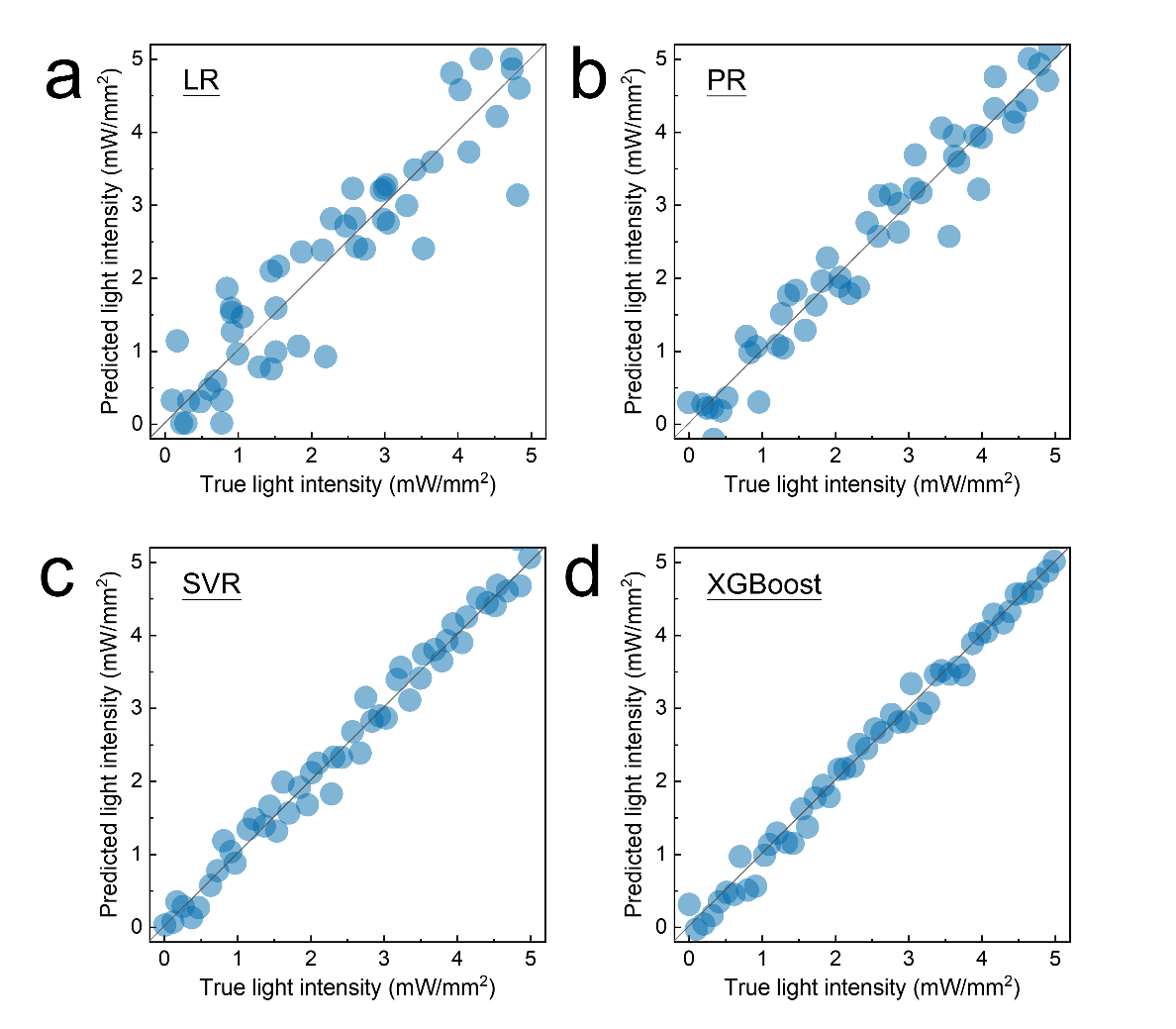


**Fig. S23** Parity plots of light intensity prediction (0–5 mW/mm^2^) for different regression models: **a** LR, **b** PR, **c** SVR, and **d** XGBoost. The solid line represents the ideal prediction (y = x).


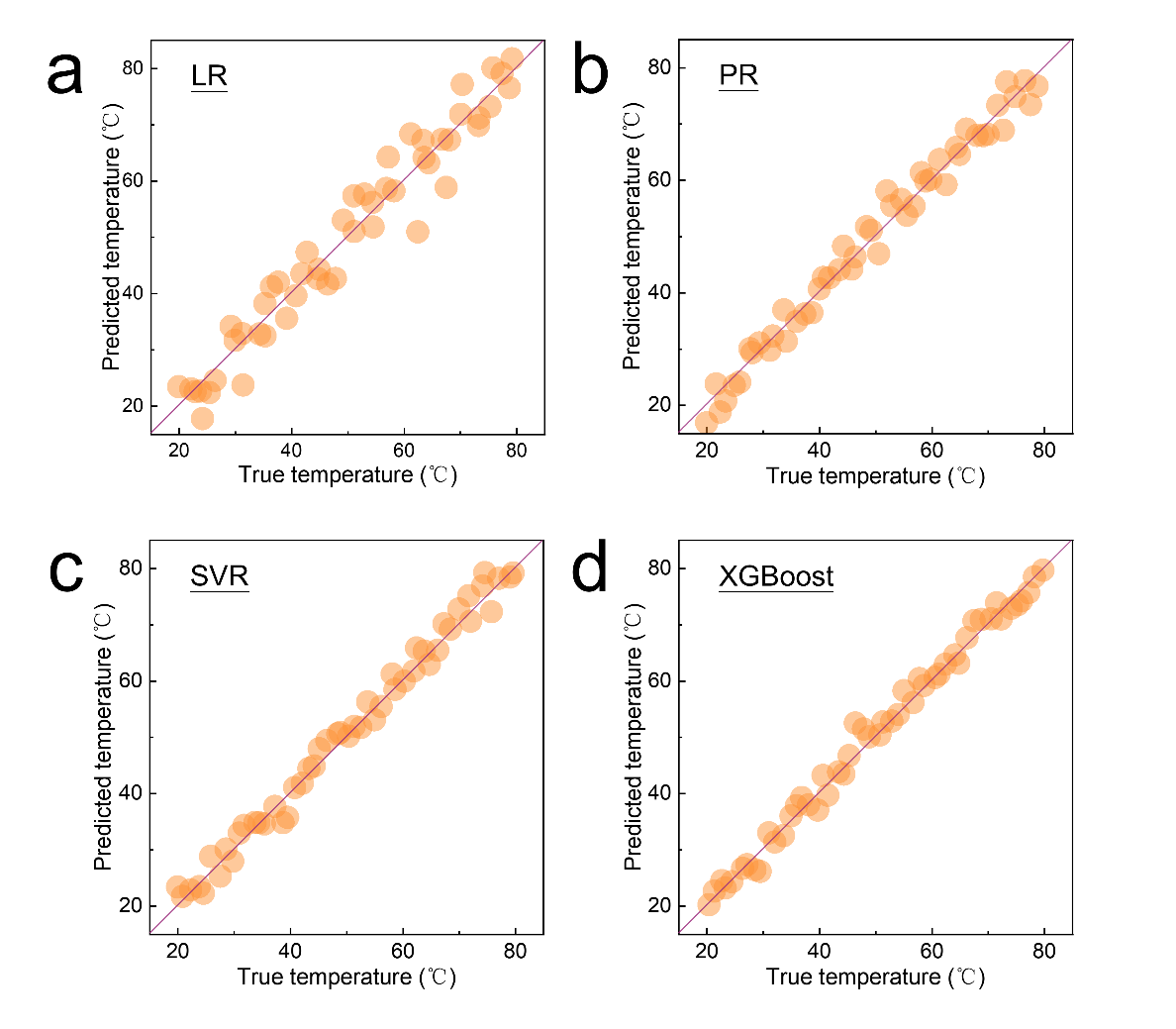


**Fig. S24** Parity plots of temperature prediction (20–80 ℃) for different regression models: **a** LR, **b** PR, **c** SVR, and **d** XGBoost. The solid line represents the ideal prediction (y = x).


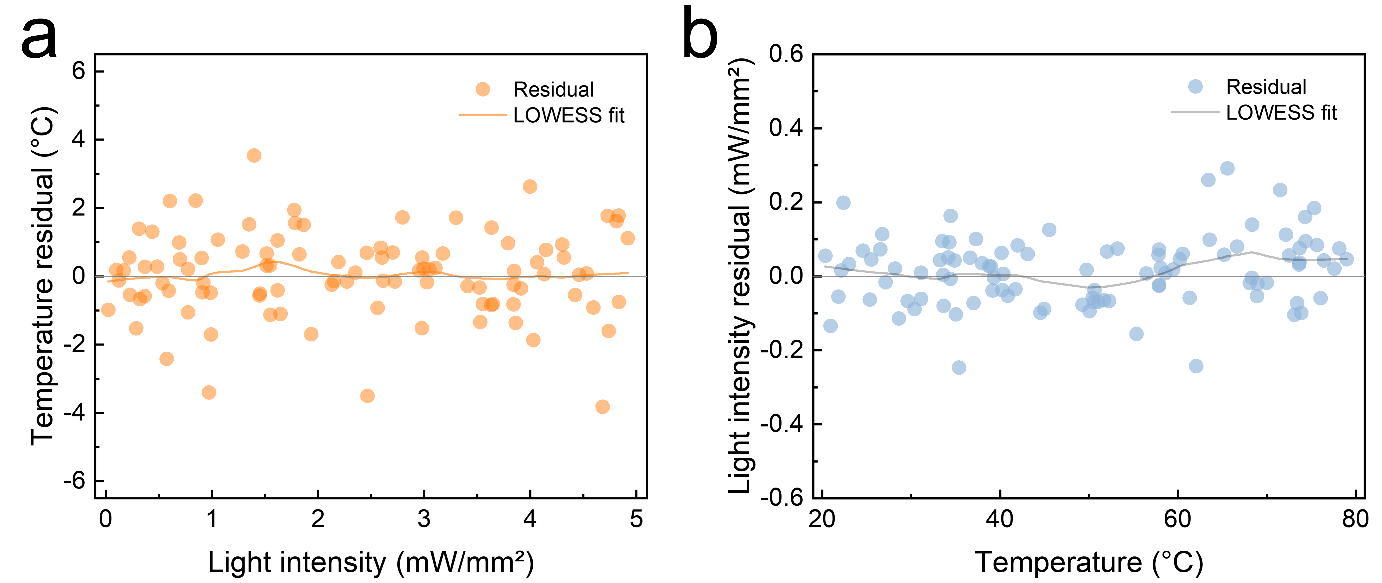
 **Fig. S25** Residual-based analysis of dual-modal prediction. **a** Temperature prediction residual as a function of light intensity. **b** Light-intensity prediction residual as a function of temperature. The residual is defined as the difference between predicted and true values. The markers represent individual test samples, and the solid lines show LOWESS fits.

**Table S1** Parameters used for calculating $D_{app}^{*}$.

| **Parameter** | **Value** |
| --- | --- |
| *V_G_* | -0.1 V |
| *P_in_* | 1 mW/cm^2^ |
| *A* | 1.5 ×10^-3^ cm^2^ |
| *I_dark_* | 4.17 ×10^-11^ A |
| *I_light_* | 4.30 ×10^-8^ A |
| *i_rms_* | 1.78 ×10^-11^ A |
| B | 3.07 Hz |

**Table S2** Mathematical models and governing equations used in the FEA simulation

| **Category** | **Physical model** | **Mathematical expression** |
| --- | --- | --- |
| Governing equations | Poisson’s equation | $\nabla\cdot\left( \varepsilon_{r}\varepsilon_{0}\nabla V \right) = 0$ |
| Boundary conditions | Surface charge | $n\cdot\left( D_{\mathrm{ZnO}}-D_{\mathrm{air}} \right) = \sigma_{\mathrm{surf}}$ |
|  | Total modulation | $\sigma_{\mathrm{surf}} \approx\sigma_{\mathrm{dark}} + \Delta\sigma_{\mathrm{light}}\left( P \right) + \Delta\sigma_{\mathrm{heat}}\left( T \right)$ |
| Phenomenological  models | Light dependence | $\Delta\sigma_{\mathrm{light}} = k_{L}\left( 1-e^{-P/P_{0}} \right)$ |
|  | Temperature dependence | $\Delta\sigma_{heat} = k_{T}\left[ e^{-E_{ads}/\left( k_{B}T \right)} - e^{-E_{ads}/\left( k_{B}T_{0} \right)} \right]$ |

**Table S3** Performance comparison of representative white-light photodetectors

| **Ref.** | **Responsivity (A/W)** | **Rise time**  **(ms)** | **Decay time**  **(ms)** |
| --- | --- | --- | --- |
| [53] | 0.9 | 153 | 164 |
| [54] | 0.1858 | 8800 | 9500 |
| [55] | 2.6×10^-2^ | 320 | 330 |
| [56] | 2.85×10^-6^ | 4000 | 4000 |
| [57] | 3.4×10^-2^ | 7210 | 6230 |
| [58] | 0.14613 | 16 | 112 |
| [59] | 10^-3^ | 180 | 120 |
| [60] | 0.7493 | 162 | 162 |
| [61] | 1.523×10^-3^ | 276 | 278 |
| [62] | 3.86×10^-2^ | 150 | 150 |
| [63] | 9.6401×10^-7^ | 200 | 400 |
| This work | 2.69 | 83 | 110 |

**Table S4** Performance comparison of representative flexible temperature sensors.

| **Ref.** | **Temperature range** | **Sensitivity (\|TCR\|)** | **Resolution** | **Response time** | **Recovery time** | **Bending cycles** |
| --- | --- | --- | --- | --- | --- | --- |
| [70] | 50 K | 1.27 %/K | 0.2 K | 0.5 s | 0.5 s | 5000 |
|  |  |  |  |  |  |  |
| [71] | 25 K | 0.77 %/K | - | 1.5 s | 6 s | 10000 |
|  |  |  |  |  |  |  |
| [72] | 50 K | 4.3 %/K | 0.1 K | - | - | 1000 |
|  |  |  |  |  |  |  |
| [73] | 45 K | 5.194 %/K | - | 2 s | 2 s | 100 |
|  |  |  |  |  |  |  |
| This work | 60 K | 7.1 %/K | 0.1 K | 0.36 s | 1.72 s | 10000 |

**Table S5** Performance comparison of representative multi-modal sensors.

| **Ref.** | **Sensing Function** | **Sensitivity** | **Min. Detectable Stimulus & Resolution** | **Response / recovery time** | **Operating Range** | **Mechanical Robustness** | **Size** |
| --- | --- | --- | --- | --- | --- | --- | --- |
| [24] | Light | 0.07 μA/(mW∙mm-2) (635 nm) | 0.13 mW·mm⁻² | ≈40 ms (optical) | 0–38 mW·mm⁻² | 8000 bending cycles  (r = 5 cm) | 0.007 mm² |
|  | Temperature | 1.79 %/K | 0.1 K | - | 300–313 K |  |  |
|  |  |  |  |  |  |  |  |
| [25] | Light | Nonlinearity (sun) | - | - | 0 – 25,000 lx | - | - |
|  | Temperature | Nonlinearity | - | - | 300–460 K |  |  |
|  | Humidity | Nonlinearity | - | ~60 s / ~70 s | 30–90 %RH |  |  |
|  |  |  |  |  |  |  |  |
| [26] | Light | 0.02 dB/Lux (sun) | - | - | 700–1900 lux | - | 846 mm² |
|  | Temperature | 0.63 dB/K | - | - | 278–298 K |  |  |
|  | Humidity | 2 MHz/%RH | - | - | 10–50 %RH |  |  |
|  | CO₂ | 0.012 dB/ppm | - | - | 800–1600 ppm |  |  |
|  |  |  |  |  |  |  |  |
| [27] | Light | - | - | - | - | 300% strain  ( 6 cycles) | - |
|  | Temperature | 0.165 μA/K | - | - | 299–333 K |  |  |
|  |  |  |  |  |  |  |  |
| [28] | Light | 0.201 μA·cm²·mW⁻¹ (980 nm) | - | 35.7 s / 33.3 s | 103.9 – 1039.5 mW·cm⁻² | - | 100 mm² |
|  | Temperature | 16.77 μA·K⁻¹ | 0.5 K | 11.9 s / 12.3 s | ΔT: -6.3 to +4.1 K |  |  |
|  |  |  |  |  |  |  |  |
| [29] | Light | 36 µA·W⁻¹ (365 nm) | 0.05 W·cm⁻² | 15.16 s / 18.65 s | 0.05 – 0.24 W·cm⁻² | - | 0.85 cm² |
|  | Temperature | 4.89 μA/K | 1.9 K | 40.55 s / 39.33 s | ΔT: −9 to +9.5 K |  |  |
|  |  |  |  |  |  |  |  |
| [30] | Light | 0.007 mA·cm²·W⁻¹ (365 nm) | - | ~101 s / 113 s | 83.6–247.8 mW·cm⁻² | - | 100 mm² |
|  | Temperature | 0.08 µA·K⁻¹ | - | Cooling: ~12 s / 5 s  Heating: ~15 s / 9.5 s | ΔT = −10.5 to +17.9 K |  |  |
|  |  |  |  |  |  |  |  |
| [31] | Light | Nonlinear (808 nm) | - | - | 0.32 – 5289.5 mW·cm⁻² | - | 1 mm² |
|  | Temperature | 894.7 μC·m⁻²·K⁻¹ | 0.1 K | - | ΔT = 0.1–27.3 K |  |  |
|  |  |  |  |  |  |  |  |
| This work | Light | 2.69 A·W⁻¹ (white light) | 1 mW·cm⁻² | 83 ms / 110 ms | 0–500 mW·cm⁻² | 10000 bending cycles (r = 2 cm) | 0.15 mm² |
|  | Temperature | 7.1 %/K | 0.1 K | 0.86 s / 1.72 s | 293–353 K |  |  |

**Table S6** Performance of different bias combinations for light–temperature decoupling

| **Bias combination** | **Light R^2^** | **Temp. R^2^** | **Light MAE (mW/cm²)** | **Temp. MAE (℃)** |
| --- | --- | --- | --- | --- |
| Single (−3.3 V) | 0.57 | 0.49 | 1.02 | 7.10 |
|  |  |  |  |  |
| Single (0 V) | 0.64 | 0.56 | 0.87 | 6.05 |
|  |  |  |  |  |
| Single (+3.3 V) | 0.71 | 0.63 | 0.73 | 5.02 |
|  |  |  |  |  |
| Dual (−3.3 / 0 V) | 0.83 | 0.77 | 0.39 | 2.85 |
|  |  |  |  |  |
| Dual (−3.3 / +3.3 V) | 0.81 | 0.74 | 0.44 | 3.18 |
|  |  |  |  |  |
| Dual (0 / +3.3 V) | 0.86 | 0.80 | 0.33 | 2.36 |
|  |  |  |  |  |
| Triple (−3.3 / 0 / +3.3 V) | 0.99 | 0.99 | 0.10 | 0.90 |

**Table S7** Comparison of representative IGZO-based multimodal sensing TFTs integrated with machine-learning-assisted processing

| **Ref.** | **Device platform** | **Stimulus modality** | **Features** | **Application** |
| --- | --- | --- | --- | --- |
| [74] | IGZO TFT + TENG | Light + Tactile | Synaptic current integration | Intelligent vehicle interaction |
|  |  |  |  |  |
| [75] | Mo charge-trapping IGZO TFT | Optical + Electrical | Conductance-state associative learning | MNIST pattern recognition |
|  |  |  |  |  |
| [76] | Light-activated IGZO TFT | Gas Species | Optical-response fingerprints | Gas recognition |
|  |  |  |  |  |
| [77] | p-Cu₂O/n-IGZO TFT | Gas Species | Complementary bidirectional responses | Gas discrimination |
|  |  |  |  |  |
| This work | FDST | Light + Temperature | Bias-dependent electrical fingerprints | Light-temperature reconstruction |

**Video S1** Real-time demonstration of the system undergoing “shadow–recovery” and “heating–removal” cycles

**Supplementary References**

- - - 1. Hullavarad, S. S., Hullavarad, N. V., Look, D. C., Claflin, B. Persistent photoconductivity studies in nanostructured ZnO UV sensors. Nanoscale Res. Lett. **4**(12), 1421–1427 (2009). <https://doi.org/10.1007/s11671-009-9414-7>
      2. Kang, Y., Nahm, H.-H., Han, S. Light-induced peroxide formation in ZnO: origin of persistent photoconductivity. Sci. Rep. **6**(1), 35148 (2016). <https://doi.org/10.1038/srep35148>
      3. Tonkoshkur, A. S., Lyashkov, A. Y., Povzlo, E. L. Kinetics of response of ZnO-Ag ceramics for resistive gas sensor to the impact of methane, and its analysis using a stretched exponential function. Sens. Actuators B Chem. **255**, 1680–1686 (2018). <https://doi.org/10.1016/j.snb.2017.08.171>
      4. Gherardi, S., Astolfi, M., Gaiardo, A., Malagù, C., et al. Investigating the temperature-dependent kinetics in humidity-resilient tin–titanium-based metal oxide gas sensors. Chemosensors **12**(8), 151 (2024). <https://doi.org/10.3390/chemosensors12080151>
